# Factors impacting vaccine uptake among adult Medicaid beneficiaries: a systematic literature review

**DOI:** 10.1093/haschl/qxae143

**Published:** 2024-11-12

**Authors:** Emily Moss, Amanda L Eiden, Louise Hartley, Justin Carrico, Raymond Farkouh, Sara Poston, Meghan Gabriel, Anna Hundt Golden, Alexandra Bhatti

**Affiliations:** RTI Health Solutions, Manchester M20 2LS, United Kingdom; Merck & Co., Inc., Rahway, NJ 07065, United States; Vaccine Evidence Research and Outcomes Consortium (VEROC), United States; RTI Health Solutions, Manchester M20 2LS, United Kingdom; RTI Health Solutions, Research Triangle Park, NC 27709, United States; Vaccine Evidence Research and Outcomes Consortium (VEROC), United States; Pfizer, New York, NY 10017, United States; Vaccine Evidence Research and Outcomes Consortium (VEROC), United States; GSK, Philadelphia, PA 19104, United States; RTI Health Solutions, Research Triangle Park, NC 27709, United States; RTI Health Solutions, Research Triangle Park, NC 27709, United States; Merck & Co., Inc., Rahway, NJ 07065, United States; Vaccine Evidence Research and Outcomes Consortium (VEROC), United States

**Keywords:** Medicaid, Affordable Care Act, adult vaccination, vaccine uptake, vaccine coverage

## Abstract

Vaccine uptake is suboptimal among adult Medicaid beneficiaries. To evaluate factors affecting vaccine uptake among adult Medicaid beneficiaries and/or affecting healthcare providers who vaccinate adult Medicaid beneficiaries, we conducted a systematic literature review in Embase, Medline, Database of Abstracts of Reviews of Effects, and the Cochrane Library for articles published from January 2005 through July 2022 and relevant conferences. For included studies, data were extracted on the study characteristics, patient and provider cost barriers, patient and provider perceived risks/benefits, and other barriers faced by patients and providers. Quality assessments were conducted using a checklist from the Joanna Briggs Institute. Twenty-one studies analyzed patient-related factors (14 studies) and/or provider-related factors (8 studies). Reviewed studies indicate that vaccine uptake is influenced by insurance benefit and cost-coverage policies, including cost-sharing, access to vaccination services, and vaccine education and awareness. Financial factors, including reimbursement for vaccine acquisition and administration, influence providers' vaccination practices for Medicaid beneficiaries. Our findings suggest that reducing or eliminating vaccination cost-sharing, promoting vaccine education and awareness about the importance and safety of vaccines, increasing access, and exploring reimbursement rates equivalent with other public or private insurance plans could mitigate barriers to vaccination for the adult Medicaid population.

## Introduction

Despite the public health importance of vaccination, there is suboptimal uptake of Advisory Committee on Immunization Practices (ACIP)–recommended vaccines among adults in the United States (US), especially adult Medicaid beneficiaries.^1^ The 2010 Affordable Care Act (ACA) and the 2022 Inflation Reduction Act (IRA) yielded federal policy changes aimed at improving coverage for the cost of vaccines. Intended to increase access to affordable health insurance and preventive health services, a major provision of the ACA was the expansion of Medicaid benefits to all adults aged < 65 years with lower income levels, often defined as income ≤ 138% of the federal poverty level (FPL). As of March 2023, 40 states and the District of Columbia have adopted the expansion of Medicaid under the ACA. (The 11 states that have chosen not to expand Medicaid include Texas, Wyoming, Kansas, Wisconsin, Mississippi, Tennessee, Alabama, Georgia, Florida, and South Carolina.) States that expanded Medicaid under the ACA were federally required to cover all ACIP-recommended vaccines with no out-of-pocket costs for vaccination services for newly eligible or “expansion” populations but were not required to extend the same coverage and cost-sharing benefits to traditionally eligible adults (ie, those eligible for Medicaid in their state before the ACA expansion). As a result, vaccine coverage and cost-sharing decisions for traditionally eligible adults in both expansion and non-expansion states remained a state decision. The IRA, implemented October 1, 2023, has attempted to extend coverage for all ACIP-recommended vaccinations, without cost-sharing, to traditionally eligible adults, such that all adults enrolled in Medicaid would receive this benefit.

Despite these federal efforts aimed at increasing vaccine uptake, each state has policies affecting the scope of vaccine coverage under Medicaid (eg, whether through medical or pharmacy benefit), which provider types are eligible to bill, reimbursement amounts for vaccine purchase and administration, and whether patient cost-sharing is permitted.^2^ Additional factors beyond state decisions about Medicaid coverage and cost-sharing also may influence patients' and providers' vaccination decisions, including convenience of access, vaccine awareness, and resource constraints.^3-8^ We conducted a systematic literature review (SLR) to evaluate factors that affect adult Medicaid beneficiaries' access to vaccinations and vaccination decisions and/or that affect the decisions of healthcare providers who serve adult Medicaid beneficiaries and administer vaccines.

## Materials and methods

We searched Embase, Medline, Database of Abstracts of Reviews of Effects (DARE), and the Cochrane Library on August 1, 2022 (see Tables A1-A4). Searches were restricted to studies published since January 1, 2005, to capture studies that were published before and after Medicaid expansion under the ACA and were relevant to the current policy environment. Conference abstracts not indexed in Embase were searched on August 13-14, 2022, as were relevant websites. Conference abstracts were limited to those indexed no earlier than 2020. All articles were screened against predefined inclusion and exclusion criteria (Table 1). At level 1 screening, 2 independent researchers reviewed the titles and abstracts of studies identified from the searches. At level 2 screening, full-text studies deemed relevant at level 1 were reviewed by 2 independent researchers. Disagreements were resolved by consensus or with a third independent researcher.

**Table 1.** List of criteria for the inclusion and exclusion of studies during the screening process.

Criterion	Included	Excluded
Population	Adult (aged >18 years) Medicaid patients and providers serving Medicaid patients in the US Low-income adults	Any populations other than Medicaid (eg, Medicare patients or providers) Pediatric patients
Intervention/comparators	*Haemophilus influenzae* type b (Hib) Hepatitis A (HepA) Hepatitis B (HepB) Human papillomavirus (HPV) Influenza Measles, mumps, and rubella (MMR) Meningococcal serogroups A, C, W, Y (MenACWY) Meningococcal serogroup B (MenB) Pneumococcal (PPSV23, PCV13) Tetanus and diphtheria (and acellular pertussis) (Td/Tdap) Varicella Herpes zoster, recombinant (RZV)	Any other vaccines
Outcomes	Patients Patient access to vaccination Costs to patient of getting a vaccine Financial cost to patient getting a vaccine (eg, out-of-pocket payment) Indirect costs to patient of getting a vaccine (eg, travel costs) Perceived benefits/risk to patient of getting a vaccine Perceived vs actual costs to patients getting a vaccine Socioeconomic barriers to vaccination Social determinants of health Providers Costs to a provider for providing a vaccine Perceived benefits to providers of providing a vaccine Perceived vs actual costs to providers of providing a vaccine Any other barriers, such as time, prioritization of other tasks, or any other nonmonetary barrier	Any other outcomes
Study design	All prospective and retrospective study types, other than those listed in the exclusions Systematic reviews (including meta-analyses)^a^	Case studies Case series Commentaries Letters
Language	English language	Non-English languages
Date	Database searches: 2005-present Conference abstract searches: past 2 years	Database searches: before 2005 Conference abstract searches: before 2020

If it was unclear whether a study met any criterion during the level 1 screening process, the study was progressed to full-text screening to confirm its inclusion in the review.

Abbreviation: US, United States.

^a^Systematic reviews were included at level 1 screening, used for identification of primary studies, and then excluded at level 2 screening.

Data were extracted from full-text articles into predefined table shells by 3 researchers and quality-checked against source documents by an independent researcher not involved in the extractions. Data on study characteristics, patient and provider cost barriers, patient and provider perceived risks/benefits, and other barriers faced by patients and providers were extracted and categorized into themes that emerged based on factors influencing vaccination. Quality assessments were performed for all included publications using the relevant checklist from JBI (formerly the Joanna Briggs Institute).^9^

## Results

### Literature search results

**Figure 1.**
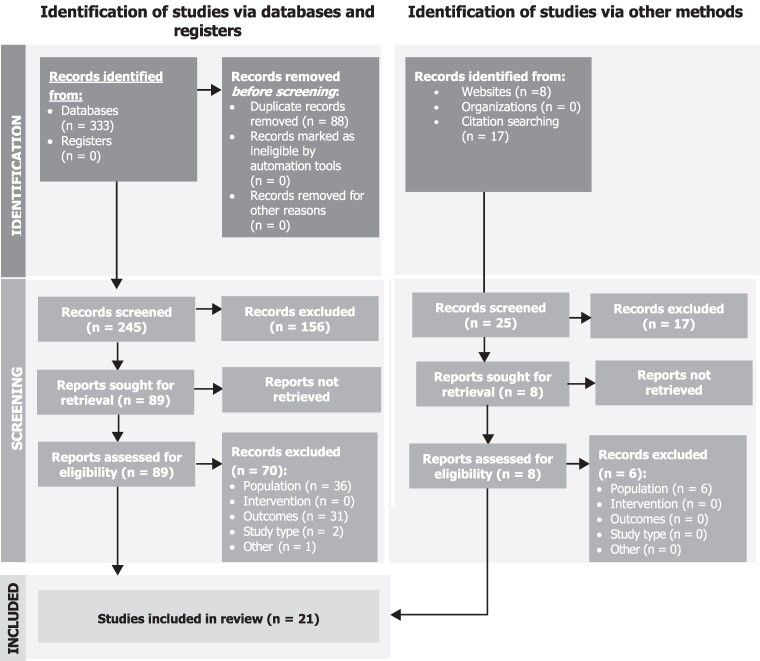
PRISMA diagram. PRISMA, Preferred Reporting Items for Systematic Reviews and Meta-Analyses. Source: PRISMA diagram adapted from Page et al.^10^

Of the 270 abstracts selected for level 1 screening (database searches = 245, internet searches = 8, reference list reviews/hand searches = 17), 97 publications progressed to level 2 screening (database searches = 89; internet searches = 1; hand searches = 7), at which 27 publications met the inclusion criteria and were selected for data extraction. At the data-synthesis stage, an additional 6 studies were excluded, resulting in 21 studies included in the review (Figure 1).

### Factors impacting vaccination among adult Medicaid beneficiaries

Fourteen studies analyzed patient-specific factors for initiating vaccination or series completion for adult Medicaid beneficiaries (Table 2). Of note, Medicaid coverage was not uniform across states, and beneficiaries may face varying potential barriers to vaccination, depending on state and local context. Although all states provide some vaccine coverage for Medicaid beneficiaries, not all states covered all ACIP-recommended vaccines for all Medicaid enrollees at the time of this review.

**Table 2.** Summary of outcomes: patient-focused studies.

Reference	Geography	Vaccine(s)	Summary of outcomes
Aris et al.^11^	Nationwide	Tdap, HZ, pneumococcal	Authors suggested that encouraging preventive care visits and identifying any barriers to vaccination at the preventive care visits, as well as assessing vaccination status at nonpreventative care appointments and improving support to providers who do not traditionally perform vaccinations, could help to increase adult vaccination rates.
Bloodworth et al.^12^	Michigan	Influenza	Despite the introduction of the ACA, cost-sharing/copayments remain a barrier for low-income individuals. Medicaid providers still experience difficulty enrolling and retaining low-income patients. Although introduction of the ACA increased utilization of the influenza vaccine from ∼39% in 2009 to ∼44% in 2014, most Medicaid beneficiaries still did not receive the vaccine.
Brown et al.^13^	Texas	Influenza, pneumonia, HPV	As part of the intervention evaluated in this study, participants received vaccine vouchers; the most commonly reported reasons for not redeeming these vouchers were lack of time and indecision.
Hatch et al.^14^	10 Medicaid expansion states and 4 non-expansion states	HPV, influenza	Beyond healthcare access, patients served by CHCs may experience additional barriers to receipt of preventive care, including factors measured (eg, race/ethnicity, income, and number of visits) and not measured (eg, health literacy, transportation, neighborhood characteristics, and history of trauma) by this study.
Hawkins et al.^15^	Maine, New Hampshire, Massachusetts	HPV	ACA policies (dependent care coverage, HPV vaccination coverage without cost-sharing, and Medicaid expansion in Massachusetts and New Hampshire) and the ACIP recommendation for routine HPV vaccination of males were all associated with increased HPV vaccination uptake for Medicaid-insured males among the analytic sample of people aged 9-26 years. However, uptake was lowest in young adults (aged 20-26 years), and uptake in this age group decreased over the study period.
Hurley et al.^16^	Nationwide	All adult vaccines	Physicians who routinely recommended vaccines reported that up to 30% of adult patients defer or refuse certain vaccines for financial reasons (eg, out-of-pocket cost or insufficient insurance coverage) in a given month. Patients covered by some state Medicaid programs may still be experiencing incomplete coverage and/or prohibitive beneficiary cost-sharing.
Hurley et al.^17^	Colorado	Influenza, Td/Tdap, pneumococcal	Centralized reminder/recall interventions were not effective at improving adult vaccination rates in a Medicaid ACO. Authors noted that staff perceived barriers to improved vaccination rates, including lack of availability of appointments (after being recalled) and adults receiving only the influenza vaccine when other vaccines were needed. Although the Standards for Adult Immunization Practice call for assessing immunization needs at each visit, authors noted that provider implementation of this standard is variable.
Kim and Zhao^18^	17 Medicaid expansion states and 19 non-expansion states	Influenza	For Medicaid expansion states, modest improvements in health insurance coverage rates and time since last routine checkup were observed relative to non-expansion states. Increases in influenza vaccination rates were not different between Medicaid expansion and non-expansion states.
Lindley et al.^19^	Nationwide	Vaccines routinely recommended for adults ≥ 19 years other than influenza (excluding travel vaccines), seasonal influenza	Lack of vaccine awareness, other issues taking precedence during short medical appointments, and physicians not recommending vaccines were listed as common patient barriers to vaccination.
New et al.^20^	California	Tdap	The study explored reasons for suboptimal Tdap uptake during pregnancy in California by focusing on mothers whose infants developed pertussis in 2016. Key barriers identified include vaccine opportunity (ie, receiving a recommendation or referral during a scheduled prenatal appointment, and being offered vaccination onsite), healthcare insurance (ie, privately insured mothers were more likely to receive Tdap than mothers insured by Medicaid), vaccine access (ie, vaccine being stocked onsite), and personal reasons.
Okoro et al.^21^	Nationwide	Influenza	Primary source of insurance, Medicaid expansion status, income, and continuity of health insurance coverage were all factors influencing the estimated prevalence of influenza vaccination in adults aged 18-64 years.
Orenstein et al.^22^	Nationwide	All adult vaccines	Authors suggested that improved Section 317 funding for adult immunization, coverage of all ACIP-recommended vaccines for state Medicaid programs, and vaccines for uninsured adults program could improve access for low-income, underinsured, and uninsured adults.
Osazuwa-Peters et al.^23^	Nationwide	HPV	Odds of HPV vaccination increased post-ACA for individuals with private insurance and Medicaid. Authors suggested that gains were driven by an increase in vaccination-enabling factors, such as decreased uninsured rates and increased regular physician visitation.
Stewart et al.^24^	Nationwide^a^	All adult vaccines	Although coverage of adult vaccines has improved from 2003 to 2012, gaps in coverage from state Medicaid programs remain, and many programs still allow cost-sharing. The least frequently covered vaccines were HPV, varicella, and herpes zoster. Even though programs responded that an ACIP recommendation was the most significant factor when determining whether to cover a vaccine, authors noted that financial considerations ultimately have the greatest impact on coverage decisions.
Stoecker et al.^25^	Nationwide^b^	Influenza	Authors found that benefit policies that may impose financial barriers led to lower vaccination uptake among all adults. Providing benefit coverage had the strongest effect on vaccine uptake, and there were statistically significant decreases in vaccine uptake among all adults when states charged larger copayments. After imputing Medicaid enrollment status, no statistically significant differences were observed for the effect of any benefit policies on vaccine uptake. The larger point estimates for Medicaid-enrolled pregnant women may indicate that they are more sensitive to additional financial barriers.
Wheldon et al.^26^	Nationwide	HPV	Although not exclusive to the Medicaid population, the study demonstrated that a key barrier to HPV vaccination across survey respondents was knowledge and education. Most respondents agreed that they did not have enough information to decide whether to receive the HPV vaccine and that they needed more information on vaccine efficacy before making a decision.
Yue et al.^27^	Nationwide	Influenza	Overall, the expansion of Medicaid helped remove some general healthcare access barriers. However, the impact was not demonstrated by the overall drop in influenza vaccine uptake, with Black and Hispanic groups seeing a larger drop in influenza vaccine uptake.

Tables AS5 and A7, respectively, in the Appendix present the study characteristics and detailed outcomes for the patient-focused studies.

Abbreviations: ACA, Affordable Care Act; ACIP, Advisory Committee on Immunization Practices; ACO, accountable care organization; CHC, community health center; HPV, human papillomavirus; HZ, herpes zoster; Td, tetanus and diphtheria; Tdap, tetanus, diphtheria, and pertussis.

^a^Two programs (WV, WI) declined to participate and 7 (IL, KS, NH, NC, OH, PA, RI) did not respond.

^b^Forty-nine states, excluding the District of Columbia and Florida.

#### Vaccine coverage policies

A notable effect on vaccination coverage was observed for adult Medicaid beneficiaries following ACA expansion.^2,12,14,18,21,23-25^ For example, Stewart et al.^24^ found that in 2012, 98% (50/51) of all state Medicaid fee-for-service arrangements covered ≥ 1 ACIP-recommended vaccine for adults, an increase from 2003 when 94% (47/50) of programs covered ≥ 1 vaccine; 71% of programs (36/51) covered all ACIP-recommended vaccines, an 8-percentage-point increase from 2003 (63%; 32/50). Further, the ACA's influence extended to coverage and benefit determinations. In a survey of 42 Medicaid program administrators, 31 ranked ACIP recommendations as the most or second-most influential factor when determining vaccine benefit coverage decisions.^24^ However, despite the improvements, gaps still existed in vaccination coverage among state Medicaid programs: Granade et al.^2^ noted that, in 2018-2019, only 22/51 programs covered all 13 ACIP-recommended vaccines to all relevant adult Medicaid beneficiaries under both fee-for-service and managed care arrangements.

Evidence on whether Medicaid expansion yielded significant improvements in adult vaccination coverage rates is mixed. Hatch et al.^14^ found that, despite improvements in insurance enrollment rates with the ACA, the absolute improvement (difference in differences [DiD]) of human papillomavirus (HPV) vaccine uptake between expansion states and non-expansion states, pre-ACA (in 2012-2013) and post-ACA (in 2014-2015), was not significantly different (1.70; 95% CI, −0.09 to 3.38; *P* = 0.062). (In this 2021 study, 28.1% of the population was aged 12-26 years, 28.5% aged 27-39 years, and 43.3% aged ≥ 40 years. Human papillomavirus vaccination was first recommended in 2019 by ACIP for all individuals aged ≤ 26 years and as a shared clinical decision-making vaccine for adults aged 27-45 years; as such, the results may not be entirely reflective of the adult population, particularly because those aged 12-20 years already had first-dollar-coverage access pre-ACA.) In contrast, a nationwide 2011-2017 survey study observed a 43% increase in HPV vaccination post-ACA (odds ratio [OR] = 1.45; 95% CI, 1.24-1.70; *P* < 0.001) and a significant increase in those receiving ≥ 2 HPV vaccine doses (OR = 1.62; 95% CI, 1.47-1.79; *P* < 0.001) for women aged 18-26 years and for those under Medicaid (OR = 1.81; 95% CI, 1.35-2.43; *P* < 0.001); the study did not provide results separately for expansion and non-expansion states.^23^

Hatch et al.^14^ also reported that influenza vaccination significantly increased in Medicaid expansion states compared with non-expansion states after the implementation of ACA (adjusted absolute prevalence DiD: 1.98 [95% CI, 0.91-3.05; *P* < 0.001]). Stoecker et al.^25^ observed that the proportion of adult Medicaid beneficiaries reporting influenza vaccinations increased from 33.1% to 34.6% (*P* < 0.01) between 2003 and 2012. Living in a state whose Medicaid program covered the influenza vaccine was associated with a 3.6-percentage-point increase (*P* < 0.001) in likelihood of reporting being vaccinated among adults aged 19-64 years and a 6.2-percentage-point increase (*P* < 0.001) among adults aged 50-64 years. Bloodworth et al.^12^ found that, in states where Medicaid covered influenza vaccination (with and without cost-sharing), patients were significantly more likely to receive the vaccine than patients in states where Medicaid did not cover the influenza vaccination (*P* < 0.001). However, 2 additional studies found no statistically significant differences in influenza vaccine coverage between Medicaid expansion and non-expansion states.^18,21^

#### Cost-sharing and direct patient costs associated with covered vaccines

Cost-sharing requirements may exacerbate barriers to vaccinations for Medicaid beneficiaries.^21,24,25^ Stewart et al.^24^ examined vaccine cost-sharing in Medicaid (for adults; vaccines for children covered by Medicaid are free to patients through the Vaccines for Children Program) between 2003 and 2012. In 2012, 21/51 Medicaid programs prohibited cost-sharing, 25 permitted cost-sharing, and 5 programs did not have explicit cost-sharing policies for vaccinations. Cost-sharing range was $0.50-$3.40, or 5% of the allowable amount the program permits a provider to bill, in 2012; however, the study did not estimate the effect this had on the uptake. Stoecker et al.^25^ observed that Medicaid cost-sharing charges were negatively associated with influenza vaccination levels for all beneficiary groups. A $1 increase in copayments was associated with a 0.6% decline in vaccination coverage (*P* = 0.014) for adults aged 19-64 years and a 1.1% decline in coverage (*P* = 0.014) for adults aged 50-64 years. Adult low-income Medicaid beneficiaries may also experience additional financial barriers to receiving healthcare services relative to adults with higher income levels and other insurance types. For example, Okoro et al.^21^ found that adults with household income < 100% of FPL were found to have lower coverage of influenza vaccination in both Medicaid expansion states (29.5%; 95% CI, 28.2%-30.9%) and Medicaid non-expansion states (26.3%; 95% CI, 25.2%-27.4%) relative to those with income levels ≥ 100% to ≤ 400% of FPL (expansion states: 30.9%; 95% CI, 30.1%-31.7%; non-expansion states: 31.4%; 95% CI, 30.7%-32.1%) and ≥ 400% of FPL (expansion states: 41.0%; 95% CI, 40.3%-41.8%; non-expansion states: 40.6%; 95% CI, 39.9%-41.4%).

#### Healthcare system access and utilization

Convenience of access may influence vaccination rates, as increased contact with the healthcare system may promote vaccination opportunities. In a 2011-2017 nationwide survey, Osazuwa-Peters et al.^23^ found a significant increase in the odds of regular physician contact after the implementation of the ACA (OR = 1.17; 95% CI, 1.09-1.25; *P* < 0.001). A lack of convenient access to vaccination services, in contrast, may negatively impact vaccine uptake even when the costs directly associated with vaccination services are waived.^13^ For example, in a 2018 study, Brown et al.^13^ investigated whether an intervention, which included health screening with referrals and vouchers, increased access and uptake of preventive health services in a Hispanic American population comprising uninsured or Medicaid beneficiaries. Vouchers for vaccinations and cancer screening could be redeemed for free services at clinics that were funded by the Texas Public Health Department. Of the 514 included participants, 23/42 participants (54.7%) were eligible for pneumococcal vaccination, 112/281 (51.4%) were eligible for HPV vaccination, and 85/165 (51.5%) were eligible for influenza vaccination. After the intervention, only 2/23 participants who were eligible for pneumococcal vaccination used their voucher, 25/112 participants eligible for HPV vaccination used their voucher, and 19/85 participants eligible for influenza vaccination used their voucher and had the vaccination. The most commonly cited barriers to vaccination were lack of time and indecision regarding vaccination.

Despite expansion of Medicaid eligibility and covered benefits within certain states after ACA implementation, Aris et al.^11^ observed that a majority of eligible adult Medicaid beneficiaries had not had a wellness visit and had not received an influenza (84.6%), herpes zoster (61.7%), or pneumococcal (50.8%) vaccine. Additionally, greater utilization of different settings (eg, hospital, pharmacy, and physician office encounters) for vaccination was observed for Medicaid patients than commercially and Medicare-insured patients; for the herpes zoster vaccine, 85.1% of vaccines were administered at a pharmacy for Medicaid patients, compared with 40.7% for commercially and Medicare-insured patients.

#### Vaccine awareness and decision-making

Evidence suggests that adult Medicaid beneficiaries may be interested in learning more about the safety and effectiveness of vaccines before deciding to get vaccinated.^26^ In a 2021 study, Wheldon et al.^26^ explored the impact of shared clinical decision-making (ie, patient and provider working together to make a care decision) and understanding regarding HPV vaccination. Although no differences were found in reported HPV vaccination, information needs between those in Medicaid expansion states and in non-expansion states with regard to their decision to be vaccinated against HPV (adjusted OR = 1.03; 95% CI, 0.74-1.44), significant differences were found in information needs between public and private health insurance beneficiaries regarding safety, personal benefit, doctor's opinion, side effects, and risks (*P* < 0.05). With respect to preferences for who makes the primary decision for HPV vaccination—the patient, the patient and the provider (shared clinical decision-making) together, or the provider—significant differences also were observed between publicly, privately, and uninsured respondents (χ^2^ = 11.5; *P* = 0.022). Proportionally more publicly insured than privately insured respondents preferred the provider to make vaccination decisions (36.8% vs 34.2%). Shared decision-making was preferred by those who were privately insured compared with publicly insured and uninsured respondents (61.3%, 24.4%, and 14.3%, respectively).

#### Medicaid expansion and health equity and social determinants of health

The 4 studies that explored the impacts of factors relevant to health equity, including gender, race, ethnicity, and other social determinants of health, yielded mixed findings.^12,15,26,27^ One study assessing the impact of the ACA's Medicaid expansion on access to primary care among low-income adults (aged 19-64 years) found no statistically significant differences in the probability of receiving an influenza vaccination in the past 12 months with Medicaid expansion (DiD: −0.68, *P* = 0.60).^27^ Medicaid expansion was associated with a 6.24-percentage-point reduction (*P* = 0.08) in the probability of receiving an influenza vaccine in the past 12 months for non-Hispanic Black individuals, a 5.12-percentage-point reduction (*P* = 0.20) for Hispanic individuals, and a 2.20-percentage-point increase (*P* = 0.15) for non-Hispanic White individuals; however, these associations were found to not be statistically significant. In contrast, Bloodworth et al.^12^ found that Medicaid expansion was associated with significant increases in influenza vaccination rates for Asian, Hawaiian, and Pacific Islander individuals (*P* < 0.05) and for Latino individuals (*P* < 0.05), but not for Black individuals or individuals of other races and ethnicities (not further specified) compared with pre-expansion rates.

In 2021, Wheldon et al.^26^ explored decision-support needs regarding HPV vaccinations among adults aged 27-45 years. Although higher education level was the strongest factor associated with higher information needs for HPV vaccination, no significant associations were observed for any other demographic characteristic, including residence in a Medicaid expansion state. Another 2021 study found that the introduction of the ACA's preventive care provisions in 2010 and ACA-related health insurance reforms of 2014 both were associated with significant increases in HPV vaccination among males and females aged 9-26 years in the Medicaid population (*P* ≤ 0.01).^15^

### Factors impacting provider provision of vaccines to adult Medicaid beneficiaries

Eight studies analyzed factors impacting providers' provision of vaccines for adult Medicaid beneficiaries, including financial factors (eg, reimbursement for vaccine acquisition and administration) and healthcare delivery and administration policies (Table 3).

**Table 3.** Summary of outcomes: provider-focused studies.

Reference	Geography	Vaccine(s)	Summary of outcomes
Cantu et al.^28^	Texas	Pneumococcal, influenza, HZ, Td	Successful interventions implemented to improve vaccination practices included standardizing the medical assistant workflow, increasing staff access to the state vaccination registry, and increasing staff access to the EMR. Patient education slideshows were played in waiting rooms and informational handouts were available in patient rooms. Effects of interventions on improved immunization rates were difficult to assess, as other immunization initiatives were implemented during the same period. A self-administered patient questionnaire to enter previous immunization history was not successful because patients did not have enough time to complete it and/or could not remember their immunization history.
Goodman et al.^29^	Michigan	Influenza, HPV, HZ, Pneumococcal, Hep A, Hep B, Tdap	Medicaid providers face additional difficulties in receiving payments for the vaccines provided, acting as a disincentive to provide adult vaccinations. Change in eligibility of patients and complex contracts impact the likelihood of nonpayment for Medicaid vaccination claims.
Granade et al.^2^	Nationwide	Influenza, Tdap, HPV, PPSV23, Hep A, Hep B	The disparity in reimbursement rates for vaccine administration and doses between Medicaid programs was a barrier because, for some programs, it is not financially viable to provide all 13 ACIP-recommended adult vaccinations. Only 22 (43%) of Medicaid programs provided all 13 ACIP-recommended adult vaccines, leaving most programs unable to provide all 13 vaccines. Although provider focused, the study noted that not all Medicaid expansion states limited copayments through the Section 4106 incentive, leading to persistent financial barriers for Medicaid beneficiaries.
Hurley et al.^16^	Nationwide	All adult vaccines	Provider barriers included the perception that patient's insurance would not cover vaccine, the perception that patients could be vaccinated more affordably elsewhere, and dissatisfaction with reimbursement. A total of 12% of reporting physicians had seriously considered stopping providing all vaccines to patients with Medicaid insurance. Authors noted that CMS may require updated information about how much it costs to immunize an adult to ensure that physician reimbursements are adequate.
Hurley et al.^17^	Colorado	Influenza, Td/Tdap, pneumococcal	Although the Standards for Adult Immunization Practice call for assessing immunization needs at each visit, authors noted that provider implementation of this standard is variable.
Lewis et al.^30^	Nationwide	PCV13, PPSV23, Tdap, Td booster, HZ, HPV	Alongside the provider costs barriers for HZ and the general barriers for both patients and providers, authors noted that health-system level barriers may include inappropriate stocking and distribution of vaccines and health centers and missing or insufficient documentation of vaccinations.
Orenstein et al.^22^	Nationwide	All adult vaccines	Although most state Medicaid programs as of 2003 covered the 5 adult vaccines recommended at the time (varicella, MMR, influenza, PPSV23, Hep B), reimbursements may not have been adequate to offset all provider costs. Recommendations from the National Vaccine Advisory Committee and Infectious Diseases Society of America note that reimbursement should be adequate to cover vaccine and nonvaccine costs.
Yarnoff et al.^7^	9 states	All adult vaccines	Medicaid reimbursements for vaccine administration and purchase were lower than reimbursements from other payer types and were sometimes insufficient to offset provider costs. Authors noted that providers may be able to increase income by better implementing processes, such as standing orders and provider reminders, to ensure that all ACIP-recommended vaccinations are given to patients.

Tables AS6 and A8, respectively, in the Appendix present the study characteristics and detailed outcomes for the provider-focused studies.

Abbreviations: ACIP, Advisory Committee on Immunization Practices; CMS, Centers for Medicare and Medicaid Services; EMR, electronic medical record; Hep, hepatitis; HPV, human papillomavirus; HZ, herpes zoster; MMR, measles-mumps-rubella vaccine; PCV, pneumococcal conjugate vaccine; PPSV, pneumococcal polysaccharide vaccine; Td, tetanus and diphtheria; Tdap, tetanus, diphtheria, and pertussis.

#### Vaccine acquisition and administration reimbursement

Provider satisfaction with reimbursement rates for vaccine supply and administration was mixed in 2 studies. In a nationwide survey of general internists and family physicians, Hurley et al.^16^ found that nearly one-third of physicians were either very dissatisfied (16%) or mostly dissatisfied (14%) with payment for vaccine purchase and vaccine administration (14% and 18%, respectively). In addition, 12% of providers stated that they had seriously considered stopping and 5% had stopped providing vaccines to their Medicaid patients due to financial reasons, administration fees, and/or reimbursement issues. Lewis et al.^30^ found that 319 health centers across 48 states expressed that Medicaid reimbursement amounts for vaccine dose (40%) and administration (36%) were inadequate.

Three of 5 studies evaluating impacts of reimbursement on providers' provision of vaccines^2,7,17,19,29^ found that reimbursements for adult Medicaid beneficiaries' vaccinations were generally lower than those for adults with Medicare or private insurance and often were lower than typical vaccine list prices.^2,7,19^ For example, Yarnoff et al.^7^ found that median Medicaid reimbursement per dose was lower than the self-reported price providers had paid per dose for 5/14 different vaccines. Granade et al.^2^ observed that, for 51 Medicaid programs, reimbursement for vaccine supply purchases was below the private sector price reported by manufacturers to the CDC for 7/13 ACIP-recommended adult vaccines. The largest observed disparities between median Medicaid reimbursement rates and private sector prices were for varicella, 9vHPV, and Tdap; the 9vHPV vaccine had the largest per-dose vaccine supply purchase reimbursement range (median, $204.87; range, $5.27 [Missouri] to $491.38 [Mississippi]). In a study surveying 104 general internal medicine and family practices, Lindley et al.^19^ found that 60% of practices noted that Medicaid reimbursement was less than supply purchase price.

Less-than-adequate Medicaid reimbursements for adult vaccination services also may result in financial losses for providers. Lindley et al.^19^ found that 55% of practices reported losing money administering vaccines to adult Medicaid patients. When asked to quantify reimbursement ranges for the first vaccine given in a visit (< $11, $11-$17, $18-$24, or > $24), 54% of practices reported receiving < $11 for Medicaid reimbursement; for other payer types, practices reported that 15%-22% of reimbursements were < $11. State-level variations in Medicaid reimbursement for vaccine administration have also been observed; Granade et al.^2^ observed that the vaccine administration reimbursement for the first dose of injected vaccine at a healthcare visit had a median reimbursement of $13.62 (range, $3.72 [South Carolina] to $28.18 [Alaska]). Yarnoff et al.^7^ analyzed the profitability of adult vaccination among 13 provider practices (states unspecified) and found that median Medicaid reimbursements for vaccine administration ($13.56) were lower than median Medicare ($20.18) and private payer ($24.57) reimbursements.

A 2019 study analyzed claims for more than 200,000 vaccines (commercial = 205 458; Medicaid = 13 126).^29^ Reimbursement occurred more frequently with private insurance (97.9%) than with Medicaid (91.6%). The most common reasons for denial of Medicaid claims were contractual arrangements (52.3%) and health plan eligibility (37.0%). For Medicaid reimbursement of vaccine administration claims, payment rates were higher for claims in a pharmacy setting (100%) than claims in a physician office setting (89.2%).

#### Healthcare delivery and administration policies

Healthcare practices and policies, such as lack of resources and incentives, may influence providers' provision of adult vaccines.^17,28^ Cantu et al.,^28^ after implementing a quality improvement project to improving vaccination rates among Medicaid, low-income, and uninsured patients, found that interventions to improve patient awareness, vaccination workflow and timelines, and access to vaccination recordkeeping were associated with increased uptake for pneumococcal (baseline, 38.9%; postintervention, 88.2%) and influenza (baseline, 55.5%; postintervention, 90.7%) vaccines.

There also may be missed opportunities to vaccinate during healthcare encounters for the Medicaid population.^17,20^ Hurley et al.^17^ found that, among 1,275 Medicaid patients aged 19-64 years who needed both influenza and tetanus, diphtheria, and acellular pertussis (Tdap) vaccinations and received any vaccine, only 5% of participants received both the Tdap and influenza vaccines at the first vaccine visit, 80% received only influenza, and 15% received only Tdap; the authors suggested that during healthcare visits, beneficiaries' vaccination records are inconsistently checked, creating missed opportunities to vaccinate. Similarly, New et al.^20^ examined vaccination rates for maternal Tdap in California. The study involved interviewing mothers of 114 infants with pertussis aged < 4 months, with illness onset during 2016, finding that Medicaid-insured mothers (*n* = 34) were significantly less likely to receive the maternal Tdap vaccination (relative risk [RR] = 0.4; 95% CI, 0.2-0.8) or to have received prenatal Tdap vaccine during the ACIP-recommended timeframe, even when it was stocked at their prenatal clinic, than those with private insurance (RR = 0.5; 95% CI, 0.3-1.1).

Reminder and recall systems, although potentially useful in promoting vaccine uptake, were found by Hurley et al.^17^ not to improve vaccination uptake for adult Medicaid beneficiaries in a regional managed care organization. Authors noted multiple perceived barriers to uptake, including lack of availability of appointments for patients after being recalled, lack of standing orders (only 1/6 healthcare entities in the study had standing orders for all 3 studied vaccines [influenza, pneumococcal, Tdap]), and lack of adult vaccination history in immunization information systems, which are more regularly populated with pediatric versus adult vaccination information.

## Discussion

This is the first published SLR designed to identify factors, such as vaccine coverage, cost-sharing, access, vaccine awareness, and resource considerations that influence vaccine uptake for adult Medicaid beneficiaries and the provision of vaccinations by their healthcare providers. Recognizing that there is interplay among these factors, the reviewed evidence offers insights that can inform policy design, intervention strategies, and initiatives to target vaccination barriers and increase coverage for Medicaid beneficiaries.

Previous studies have analyzed the determinants of vaccination in both pediatric and adult populations. For instance, Thomson et al.^31^ proposed the 5As taxonomy (access, affordability, awareness, acceptance, and activation) for categorizing factors associated with pediatric and adult vaccine uptake and systematically reviewed evidence in each domain. Access, affordability, and awareness were factors influencing vaccine uptake among adult Medicaid beneficiaries in the studies we reviewed. Similarly, a 2022 global systematic review on vaccination among older adults found that provider recommendations, patient and provider awareness, health literacy, socioeconomic status and education, convenience of access, and healthcare consumption all influence vaccine uptake.^32^ Our review found insurance benefit and coverage, including cost-sharing policies; access to vaccination services; and vaccine awareness to be factors affecting vaccination uptake for adult Medicaid beneficiaries. Although our focus was on factors affecting access to vaccination and vaccination decisions of adult Medicare beneficiaries and their healthcare providers, vaccine hesitancy also plays an important role in decision-making and may warrant further exploration in this population.

Medicaid expansion under the ACA has improved access to and coverage for care, with a generally favorable impact on vaccination patterns for the 55 million adult Medicaid beneficiaries. Given other documented benefits of Medicaid expansion, such as increased access to care,^33-35^ that may improve vaccination uptake, this finding suggests that the ACA has positively influenced vaccination rates for Medicaid beneficiaries. In addition, the recent passage of the IRA was intended to eliminate cost-sharing for ACIP-recommended vaccines for Medicaid beneficiaries in expansion states, potentially further improving uptake.

Although cost-sharing requirements may present barriers to vaccinations for Medicaid beneficiaries,^25^ reduced costs for vaccination services and support for indirect costs associated with vaccine encounters (eg, transportation and childcare), especially for lower-income populations, may also promote vaccination uptake. Convenience of access is another crucial factor, and ensuring that all healthcare encounters, including wellness and acute care visits, provide opportunities for vaccination of Medicaid beneficiaries is essential.^11,17,20^

To promote convenience of access, pharmacy vaccination for Medicaid beneficiaries may be of value. Pharmacies and pharmacists hold a position of trust and accessibility within communities, which is particularly valuable in rural and underserved areas.^11,36,37^ However, the extent to which Medicaid beneficiaries utilize pharmacies for vaccinations varies by vaccine.^11^ To enhance vaccine uptake and confidence, there is a need to increase clinical capacity, improve reimbursement policies, and raise awareness regarding pharmacies' role in vaccine administration for Medicaid beneficiaries.^38^

Provider-related factors (eg, reimbursement policies) also affect vaccine uptake among Medicaid beneficiaries, and variations in coverage for different provider arrangements also may influence Medicaid beneficiaries' vaccine uptake.^39^ The potential impact of denial of Medicaid claims on providers' and patients' future vaccination behavior may warrant further study. Reimbursement challenges, in addition to capacity challenges and time limitations, may also impact prioritization of vaccines.^19^ Therefore, any factors contributing to provider burden, financial or otherwise, warrant consideration in the broader discussion of vaccine administration. Providers also may face challenges arising from frequent updates to ACIP recommendations.^19,30^ These challenges result in providers not consistently or appropriately recommending the full schedule of ACIP-recommended adult vaccinations, highlighting the need for a streamlined system to support vaccination practices.

The insights gained from this SLR may help to inform strategies targeting barriers to vaccination and increasing vaccine uptake for Medicaid beneficiaries. Policy initiatives, such as the IRA, seek to close general gaps in coverage for adults eligible for Medicaid, and explorations are warranted into the impact of the IRA incentive to provide first-dollar coverage to adult Medicaid beneficiaries by the temporary increase in federal matching of states' funds. These policy measures reflect a concerted effort to bridge gaps in access to essential vaccines and promote public health. Although first-dollar coverage is critical, convenient and equitable access also will mitigate the remaining barriers to uptake among adult Medicaid beneficiaries. Further, social determinants of health may hinder vaccine access and uptake, and additional research should explore how they impact uptake to better inform policy and programmatic discussions. Awareness among adult Medicaid beneficiaries regarding recommended vaccines may be limited for a subset, and this may be compounded by changing coverage policies and vaccination guidelines.

Limitations of this SLR are noted. Additional factors influencing vaccination decisions may not have been directly addressed in the studies identified. The review was not designed to identify which vaccines were and were not covered by Medicaid; vaccine-specific coverage and uptake patterns for Medicaid beneficiaries are not reflected here but have been explored in previous research.^40^ Studies evaluating COVID-19 vaccination decisions were not evaluated because the environmental circumstances were different than for routine vaccination. Information was found for only some US states, which may differ in vaccination practices and policies, political affiliations, demographics, and other unobserved characteristics. Finally, some studies included Medicaid patients or providers in the study population but did not stratify results by Medicaid status; therefore, findings from such studies could not be attributed specifically to Medicaid beneficiaries or providers.

## Conclusions

Many complex factors influence vaccine uptake for adult Medicaid beneficiaries. Reducing or eliminating patient out-of-pocket costs, providing accurate and appropriate information about the importance and safety of vaccines, increasing convenience, and exploring reimbursement rates equivalent with other public or private insurance plans could mitigate barriers and help improve equitable vaccine access for Medicare beneficiaries.

## Supplementary Material

qxae143_Supplementary_Data

## Data Availability

All relevant data are included within the manuscript and its supplementary materials.
