# Supplementary material for: Factors impacting vaccine uptake among adult Medicaid beneficiaries: a systematic literature review
Source: Health Aff Sch. 2024 Nov 12;2(11):qxae143. doi: 10.1093/haschl/qxae143 (PMC11583532; doi:10.1093/haschl/qxae143)
Supplement: qxae143_Supplementary_Data [file qxae143_supplementary_data.zip › Appendix_13Nov2024.pdf]

SUPPLEMENTARY APPENDIX

Table A1. Embase Pilot Search Strategy for the Systematic Literature Review on Factors Affecting the Uptake and Provision of Vaccines (Search Conducted 1 August 2022)

Search number	Search terms	Hits
**Population**		
#1	'adult'/exp OR adult*:ti,ab,de OR 'grown-up*':ti,ab,de OR grownup*:ti,ab,de OR 'institutionalized adult'/exp OR 'middle aged'/exp OR 'middle age*':ti,ab,de OR 'middle-age*':ti,ab,de OR 'young adult'/exp OR 'aged'/exp OR aged:ti,ab,de OR elderl*:ti,ab,de OR senior*:ti,ab,de OR senium:ti,ab,de OR 'aged hospital patient'/exp OR 'frail elderly'/exp OR 'institutionalized elderly'/exp OR 'very elderly':ti,ab,de OR '80 and over':ti,ab,de OR centenarian*:ti,ab,de OR nonagenarian*:ti,ab,de OR octogenarian*:ti,ab,de OR 'very old':ti,ab,de OR 'pensioner'/exp OR pensioner*:ti,ab,de	11,416,033
#2	'medicaid'/exp OR medicaid:ti,ab,de OR 'publicly insured':ti,ab,de	65,518
#3	#1 AND #2	28,967
**Intervention**		
#4	'immunization'/exp OR immunization*:ti,ab,de OR immunisation:ti,ab,de OR ((immunostimulation NEAR/1 therap*):ti,ab,de) OR reimmunzation*:ti,ab,de OR reimmunization*:ti,ab,de OR 'active immunization'/exp OR 'genetic immunization'/exp OR 'dna immunization'/exp OR 'gene gun immunization'/exp OR 'rna immunization'/exp OR 'mass immunization'/exp OR 'secondary immunization'/exp OR 'vaccination coverage'/exp OR 'vaccine failure'/exp OR 'vaccination'/exp OR vaccination*:ti,ab,de OR vaccine*:ti,ab,de OR vaccinotherap*:ti,ab,de	660,551
#5	'haemophilus influenzae type b vaccine'/exp OR 'act hib':ti,ab,de OR 'act-hib':ti,ab,de OR acthib:ti,ab,de OR 'haemophilus b conjugate':ti,ab,de OR hbpv:ti,ab,de OR 'hib ompc':ti,ab,de OR 'hib vax':ti,ab,de OR hiberix:ti,ab,de OR hibest:ti,ab,de OR hibtiter:ti,ab,de OR omnihib:ti,ab,de OR 'pedvax hib':ti,ab,de OR pedvaxhib:ti,ab,de OR prohibit:ti,ab,de OR 'vaxem hib':ti,ab,de	9,578
#6	'hepatitis a vaccine'/exp OR avaxim:ti,ab,de OR epaxal:ti,ab,de OR havpur:ti,ab,de OR havrix:ti,ab,de OR 'mevac a':ti,ab,de OR nothav:ti,ab,de OR vaqta:ti,ab,de	5,634
#7	'hepatitis b vaccine'/exp OR fendrix:ti,ab,de OR 'gen h b vax':ti,ab,de OR 'gen hb vax':ti,ab,de OR 'h b vax':ti,ab,de OR 'hb vax':ti,ab,de OR 'hepaccine b':ti,ab,de OR hepagene:ti,ab,de OR hepatavax:ti,ab,de OR 'hepavaxx b':ti,ab,de OR heplisav:ti,ab,de OR heprecomb:ti,ab,de OR 'heptavax b':ti,ab,de OR hepuman:ti,ab,de OR 'hevac b':ti,ab,de OR supervax:ti,ab,de OR 'tgp 943':ti,ab,de OR theradigm:ti,ab,de OR 'v 270':ti,ab,de OR v270:ti,ab,de	20,940

Search number	Search terms	Hits
#8	‘Wart virus vaccine’/exp OR ceravix:ti,ab,de OR cervarix:ti,ab,de OR gardasil:ti,ab,de OR silgard:ti,ab,de OR 'hpv vaccination'/exp	16,560
#9	'influenza vaccine'/exp OR 'a-rix':ti,ab,de OR adjupanrix:ti,ab,de OR admune:ti,ab,de OR aflunov:ti,ab,de OR afluria:ti,ab,de OR aggripal:ti,ab,de OR agriflu:ti,ab,de OR agrippal:ti,ab,de OR 'alfa-rix':ti,ab,de OR 'alfa-rix-tetra':ti,ab,de OR alorbat:ti,ab,de OR 'alpha-rix':ti,ab,de OR 'alpha-rix-tetra':ti,ab,de OR anflu:ti,ab,de OR arepanrix:ti,ab,de OR batrevac:ti,ab,de OR begripal:ti,ab,de OR begrivac:ti,ab,de OR 'bvx m001':ti,ab,de OR celtura:ti,ab,de OR celvapan:ti,ab,de OR chiroflu:ti,ab,de OR chiromas:ti,ab,de OR daronrix:ti,ab,de OR enzira:ti,ab,de OR 'flu immune':ti,ab,de OR 'flu imune':ti,ab,de OR 'flu-vac':ti,ab,de OR fluad:ti,ab,de OR fluarix*:ti,ab,de OR fluax:ti,ab,de OR flublok:ti,ab,de OR flucelvax:ti,ab,de OR fluenz:ti,ab,de OR flugen*:ti,ab,de OR fluinsure:ti,ab,de OR flulaval:ti,ab,de OR flumist:ti,ab,de OR fluogen:ti,ab,de OR flushield:ti,ab,de OR flustat:ti,ab,de OR fluvaccinol:ti,ab,de OR fluvarix:ti,ab,de OR fluvax:ti,ab,de OR fluviral:ti,ab,de OR fluvirin*:ti,ab,de OR fluviron:ti,ab,de OR fluzone:ti,ab,de OR focetria:ti,ab,de OR foclivia:ti,ab,de OR gammaflu:ti,ab,de OR grippovac:ti,ab,de OR humenza:ti,ab,de OR idflu:ti,ab,de OR immugrip:ti,ab,de OR imuvac:ti,ab,de OR inflexal:ti,ab,de OR influject:ti,ab,de OR influpozzi:ti,ab,de OR influsplit:ti,ab,de OR influvac:ti,ab,de OR intanza:ti,ab,de OR 'inviron-ol':ti,ab,de OR invivac:ti,ab,de OR iradogen:ti,ab,de OR istivac:ti,ab,de OR mastaflu:ti,ab,de OR 'medi 3314':ti,ab,de OR 'medi-3314':ti,ab,de OR medi3314:ti,ab,de OR 'mfv ject':ti,ab,de OR 'multimeric 001':ti,ab,de OR munevan:ti,ab,de OR mutagrip:ti,ab,de OR nasovax:ti,ab,de OR nivgrip:ti,ab,de OR optaflu:ti,ab,de OR pandemrix:ti,ab,de OR panenza:ti,ab,de OR panvax:ti,ab,de OR 'pf 4522625':ti,ab,de OR pf4522625:ti,ab,de OR preflucel:ti,ab,de OR prepandrix:ti,ab,de OR previgrip:ti,ab,de OR pumarix:ti,ab,de OR sandovac:ti,ab,de OR serinflu:ti,ab,de OR 'skf 106160':ti,ab,de OR supemtek:ti,ab,de OR trivalent:ti,ab,de OR ultragrivac:ti,ab,de OR vacciflu:ti,ab,de OR 'vax 102':ti,ab,de OR vax102:ti,ab,de OR vaxigrip:ti,ab,de OR vaxigriptetra:ti,ab,de OR vepacel:ti,ab,de OR viroflu:ti,ab,de OR 'x-flu':ti,ab,de OR xanaflu:ti,ab,de	51,063
#10	'measles mumps rubella vaccine'/exp OR immravax:ti,ab,de OR 'm m rvaxpro':ti,ab,de OR 'm-m-r ii':ti,ab,de OR 'm-m-r vax':ti,ab,de OR 'm-m-rvaxpro':ti,ab,de OR 'm.m.r. ii':ti,ab,de OR 'mm rvaxpro':ti,ab,de OR mmr:ti,ab,de OR mmrvaxpro:ti,ab,de OR morupar:ti,ab,de OR 'mumeru vax':ti,ab,de OR pluserix:ti,ab,de OR priorix:ti,ab,de OR 'r.o.r. vax':ti,ab,de OR 'tri-kovax':ti,ab,de OR trimovax:ti,ab,de OR triviraten:ti,ab,de OR trivirix:ti,ab,de OR virivac:ti,ab,de	22,837
#11	'meningococcus vaccine'/exp OR 'acwy vax':ti,ab,de OR bexsero:ti,ab,de OR 'men quad tt':ti,ab,de OR menactra:ti,ab,de OR menacwy:ti,ab,de OR menafrivac:ti,ab,de OR menbvac:ti,ab,de OR mencevax:ti,ab,de OR meningitec:ti,ab,de OR meninvact:ti,ab,de OR menjugate:ti,ab,de OR menomune:ti,ab,de OR menpovax:ti,ab,de OR menquadfi:ti,ab,de OR menveo:ti,ab,de OR menzb:ti,ab,de OR 'neisvac c':ti,ab,de OR nimenrix:ti,ab,de OR nonamen:ti,ab,de OR 'tetramen-t':ti,ab,de OR trumenba:ti,ab,de OR 'va mengoc bc':ti,ab,de OR 'va-mengoc-bc':ti,ab,de	8,150

Search number	Search terms	Hits
#12	'pneumococcus vaccine'/exp OR moniarix:ti,ab,de OR 'pcv 13':ti,ab,de OR pcv13:ti,ab,de OR 'phid cv':ti,ab,de OR 'pneu immune':ti,ab,de OR 'pneumo 23':ti,ab,de OR 'ppv23':ti,ab,de OR pneumopur:ti,ab,de OR pneumovax:ti,ab,de OR 'pnu immune':ti,ab,de OR 'pnu imune':ti,ab,de OR 'pnu-imune 23':ti,ab,de OR prevenar*:ti,ab,de OR prevnar:ti,ab,de OR streptopur:ti,ab,de OR streptorix:ti,ab,de	22,607
#13	'diphtheria tetanus vaccine'/exp OR 'd t vax':ti,ab,de OR 'dif tet all':ti,ab,de OR diftavax:ti,ab,de OR diftetall:ti,ab,de OR ditanrix:ti,ab,de OR ditebooster:ti,ab,de OR 'dt reduct':ti,ab,de OR 'imovax d t':ti,ab,de OR 'td immun':ti,ab,de OR 'td pur':ti,ab,de OR 'td rix':ti,ab,de OR tedivax:ti,ab,de OR 'diphtheria pertussis tetanus vaccine'/exp OR 'acel immune':ti,ab,de OR 'acel imune':ti,ab,de OR adacel:ti,ab,de OR 'adsorbed dt coq':ti,ab,de OR 'anatoxal di te per berna':ti,ab,de OR ((booster NEAR/1 tdap):ti,ab,de) OR boostertdap:ti,ab,de OR boostrix:ti,ab,de OR certiva:ti,ab,de OR covaxis:ti,ab,de OR 'd.t. coq':ti,ab,de OR daptacel:ti,ab,de OR 'dif per tet all':ti,ab,de OR 'diphtheria plus pertussis plus tetanus':ti,ab,de OR 'diphtheria tetanus pertussis trivaccine':ti,ab,de OR 'diteki booster':ti,ab,de OR ditekibooster:ti,ab,de OR dpt:ti,ab,de OR dtap*:ti,ab,de OR dtp:ti,ab,de OR infanrix*:ti,ab,de OR neodiftepertus:ti,ab,de OR 'p.d.t. vax purified':ti,ab,de OR pertugen:ti,ab,de OR 'tdap-immun':ti,ab,de OR tdapbooster:ti,ab,de OR 'tri immunol':ti,ab,de OR triacelluvax:ti,ab,de OR triaxis:ti,ab,de OR tribaccine:ti,ab,de OR tripacel:ti,ab,de OR tripedia:ti,ab,de OR tripvac:ti,ab,de OR tritanrix:ti,ab,de OR trivax:ti,ab,de	15,342
#14	'chickenpox vaccine'/exp OR okavax:ti,ab,de OR suduvax:ti,ab,de OR 'v-z vax':ti,ab,de OR 'vaccin varilrix':ti,ab,de OR varipox:ti,ab,de OR varivax:ti,ab,de	5,200
#15	'varicella zoster vaccine'/exp OR shingrix:ti,ab,de OR varilrix:ti,ab,de OR zostavax:ti,ab,de	3,963
#16	#4 OR #5 OR #6 OR #7 OR #8 OR #9 OR #10 OR #11 OR #12 OR #13 OR #14 OR #15	689,127
**Outcomes**		
#17	'social determinants of health'/exp OR 'social determinant*':ti,ab,de OR 'social determining factor':ti,ab,de OR 'social health determinant':ti,ab,de OR 'social factors determining health':ti,ab,de OR 'socioeconomics'/exp OR 'economic value of life':ti,ab,de OR 'indigent health care':ti,ab,de OR 'medical indigency':ti,ab,de OR 'social economic*':ti,ab,de OR 'socio economic*':ti,ab,de OR socioeconomic*:ti,ab,de OR 'health disparity'/exp OR 'health disparit*':ti,ab,de OR 'health inequalit*':ti,ab,de OR 'health inequit*':ti,ab,de OR 'health status disparity':ti,ab,de OR 'health status disparities':ti,ab,de OR 'health status inequality':ti,ab,de OR 'health status inequalities':ti,ab,de OR 'health status inequity':ti,ab,de OR 'health status inequities':ti,ab,de OR 'poverty'/exp OR poverty:ti,ab,de OR indigence:ti,ab,de OR indigency:ti,ab,de OR impoverished:ti,ab,de OR 'low income':ti,ab,de OR 'underserved population*':ti,ab,de OR 'underserved communit*':ti,ab,de OR 'barriers':ti,ab,de	1,544,847
**Geographic limitation**		
#18	'united states'/exp OR 'united states':ti,ab OR usa:ti,ab OR 'u.s.a.':ti,ab OR 'u.s.':ti,ab OR 'united states':ad OR 'usa':ad OR 'u.s.a.':ad OR 'u.s.':ad OR	11,798,057

Search number	Search terms	Hits
	'us':ad OR alabama:ad OR alaska:ad OR arizona:ad OR arkansas:ad OR california:ad OR colorado:ad OR connecticut:ad OR delaware:ad OR florida:ad OR georgia:ad OR hawaii:ad OR idaho:ad OR illinois:ad OR indiana:ad OR iowa:ad OR kansas:ad OR kentucky:ad OR louisiana:ad OR maine:ad OR maryland:ad OR massachusetts:ad OR michigan:ad OR minnesota:ad OR mississippi:ad OR missouri:ad OR montana:ad OR nebraska:ad OR nevada:ad OR 'new hampshire':ad OR 'new jersey':ad OR 'new mexico':ad OR 'new york':ad OR 'north carolina':ad OR 'north dakota':ad OR ohio:ad OR oklahoma:ad OR oregon:ad OR pennsylvania:ad OR 'rhode island':ad OR 'south carolina':ad OR 'south dakota':ad OR tennessee:ad OR texas:ad OR utah:ad OR vermont:ad OR virginia:ad OR washington:ad OR 'west virginia':ad OR wisconsin:ad OR wyoming:ad OR 'puerto rico':ad OR alabama:de,ab,ti OR alaska:de,ab,ti OR arizona:de,ab,ti OR arkansas:de,ab,ti OR california:de,ab,ti OR colorado:de,ab,ti OR connecticut:de,ab,ti OR delaware:de,ab,ti OR florida:de,ab,ti OR georgia:de,ab,ti OR hawaii:de,ab,ti OR idaho:de,ab,ti OR illinois:de,ab,ti OR indiana:de,ab,ti OR iowa:de,ab,ti OR kansas:de,ab,ti OR kentucky:de,ab,ti OR louisiana:de,ab,ti OR maine:de,ab,ti OR maryland:de,ab,ti OR massachusetts:de,ab,ti OR michigan:de,ab,ti OR minnesota:de,ab,ti OR mississippi:de,ab,ti OR missouri:de,ab,ti OR montana:de,ab,ti OR nebraska:de,ab,ti OR nevada:de,ab,ti OR 'new hampshire':de,ab,ti OR 'new jersey':de,ab,ti OR 'new mexico':de,ab,ti OR 'new york':de,ab,ti OR 'north carolina':de,ab,ti OR 'north dakota':de,ab,ti OR ohio:de,ab,ti OR oklahoma:de,ab,ti OR oregon:de,ab,ti OR pennsylvania:de,ab,ti OR 'rhode island':de,ab,ti OR 'south carolina':de,ab,ti OR 'south dakota':de,ab,ti OR tennessee:de,ab,ti OR texas:de,ab,ti OR utah:de,ab,ti OR vermont:de,ab,ti OR virginia:de,ab,ti OR washington:de,ab,ti OR 'west virginia':de,ab,ti OR wisconsin:de,ab,ti OR wyoming:de,ab,ti OR 'puerto rico':de,ab,ti	
**Exclusion terms**		
#19	'animal'/exp NOT 'human'/exp	5,817,251
#20	comment*:ti OR 'letter':it OR 'editorial':it OR 'case report'/exp OR 'case stud*':ti OR 'case report*':ti OR 'case series':ti	4,794,214
**Total**		
#21	#3 AND #16 AND #17 AND #18	271
#22	#21 NOT (#19 OR #20)	262
#23	#22 AND ([article]/lim OR [article in press]/lim OR [erratum]/lim OR [review]/lim) AND [1-1-2005]/sd NOT [31-12-2022]/sd AND [2005-2022]/py	167
#24	#22 AND ([conference abstract]/lim OR [conference paper]/lim OR [conference review]/lim) AND [1-1-2020]/sd NOT [31-12-2022]/sd AND [2020-2022]/py	30
#25	#23 OR #24	197

Table A2. PubMed Search Strategy for the Systematic Literature Review on Factors Affecting the Uptake and Provision of Vaccines (Search Conducted 1 August 2022)

Search number	Search terms	Hits
**Population**		
#1	"Adult"[Mesh] OR adult*[Text Word] OR “grown-up*”[Text Word] OR grownup*[Text Word] OR "Institutionalization"[Mesh:NoExp] OR “middle aged”[Mesh] OR “middle age*”[Text Word] OR “middle-age*”[Text Word] OR "Young Adult"[Mesh] OR "Aged"[Mesh] OR aged[Text Word] OR elderl*[Text Word] OR senior*[Text Word] OR senium[Text Word] OR "Frail Elderly"[Mesh] OR “very elderly”[Text Word] OR “80 and over”[Text Word] OR centenarian*[Text Word] OR nonagenarian*[Text Word] OR octogenarian*[Text Word] OR “very old”[Text Word] OR pensioner*[Text Word]	8,848,861
#2	"Medicaid"[Mesh] OR Medicaid[Text Word] OR “Publicly Insured”[Text Word]	53,021
#3	#1 AND #2	19,128
**Intervention**		
#4	"Immunization"[Mesh] OR immunization*[Text Word] OR immunisation[Text Word] OR “immunostimulation therap*”[Text Word] OR reimmunzation*[Text Word] OR reimmunization*[Text Word] OR "Biolistics"[Mesh] OR "Mass Vaccination"[Mesh] OR "Immunization, Secondary"[Mesh] OR "Vaccination Coverage"[Mesh] OR "Vaccination"[Mesh] OR vaccination*[Text Word] OR vaccine*[Text Word] OR vaccinotherap*[Text Word]	526,634
#5	"Haemophilus influenzae type b polysaccharide vaccine"[Supplementary Concept] OR “act hib”[Text Word] OR “act-hib”[Text Word] OR acthib[Text Word] OR “haemophilus b conjugate”[Text Word] OR hbpv[Text Word] OR “hib ompc”[Text Word] OR “hib vax”[Text Word] OR hiberix[Text Word] OR hibest[Text Word] OR hibtiter[Text Word] OR omnihib[Text Word] OR “pedvax hib”[Text Word] OR pedvaxhib[Text Word] OR prohibit[Text Word] OR “vaxem hib”[Text Word]	4,645
#6	"Hepatitis A Vaccines"[Mesh] OR avaxim[Text Word] OR epaxal[Text Word] OR havpur[Text Word] OR havrix[Text Word] OR mevac-a[Text Word] OR nothav[Text Word] OR vaqta[Text Word]	1,823
#7	"Hepatitis B Vaccines"[Mesh] OR fendrix[Text Word] OR “gen h b vax”[Text Word] OR “gen hb vax”[Text Word] OR “h b vax”[Text Word] OR “hb vax”[Text Word] OR “hepaccine b”[Text Word] OR hepagene[Text Word] OR hepatavax[Text Word] OR “hepavaxx B”[Text Word] OR heplisav[Text Word] OR heprecomb[Text Word] OR “heptavax b”[Text Word] OR hepuman[Text Word] OR “hevac b”[Text Word] OR supervax[Text Word] OR “tgp 943”[Text Word] OR theradigm[Text Word] OR “v 270”[Text Word] OR v270[Text Word]	9,942
#8	"Papillomavirus Vaccines"[Mesh] OR ceravix[Text Word] OR cervarix[Text Word] OR gardasil[Text Word] OR silgard[Text Word]	9,708
#9	"Influenza Vaccines"[Mesh] OR “a-rix”[Text Word] OR adjupanrix[Text Word] OR admune[Text Word] OR aflunov[Text Word] OR afluria[Text Word] OR aggripal[Text Word] OR agriflu[Text Word] OR agrippal[Text Word] OR “alfa-rix”[Text Word] OR “alfa-rix-tetra”[Text Word] OR alorbat[Text Word] OR “alpha-rix”[Text Word] OR “alpha-rix-tetra”[Text Word] OR anflu[Text Word] OR	33,972

Search number	Search terms	Hits
	arepanrix[Text Word] OR batrevac[Text Word] OR begripal[Text Word] OR begrivac[Text Word] OR “bvx m001”[Text Word] OR celtura[Text Word] OR celvapan[Text Word] OR chiroflu[Text Word] OR chiromas[Text Word] OR daronrix[Text Word] OR enzira[Text Word] OR “flu immune”[Text Word] OR “flu imune”[Text Word] OR “flu-vac”[Text Word] OR fluad[Text Word] OR fluarix*[Text Word] OR fluax[Text Word] OR flublok[Text Word] OR flucelvax[Text Word] OR fluenz[Text Word] OR flugen*[Text Word] OR fluinsure[Text Word] OR flulaval[Text Word] OR flumist[Text Word] OR fluogen[Text Word] OR flushield[Text Word] OR flustat[Text Word] OR fluvaccinol[Text Word] OR fluvarix[Text Word] OR fluvax[Text Word] OR fluviral[Text Word] OR fluvirin*[Text Word] OR fluviron[Text Word] OR fluzone[Text Word] OR focetria[Text Word] OR foclivia[Text Word] OR gammaflu[Text Word] OR grippovac[Text Word] OR humenza[Text Word] OR idflu[Text Word] OR immugrip[Text Word] OR imuvac[Text Word] OR inflexal[Text Word] OR fluject[Text Word] OR influpozzi[Text Word] OR influsplit[Text Word] OR influvac[Text Word] OR intanza[Text Word] OR “inviron-ol”[Text Word] OR invivac[Text Word] OR iradogen[Text Word] OR istivac[Text Word] OR mastaflu[Text Word] OR “medi 3314”[Text Word] OR “medi-3314”[Text Word] OR medi3314[Text Word] OR “mfv ject”[Text Word] OR “multimeric 001”[Text Word] OR munevan[Text Word] OR mutagrip[Text Word] OR nasovax[Text Word] OR nivgrip[Text Word] OR optaflu[Text Word] OR pandemrix[Text Word] OR panenza[Text Word] OR panvax[Text Word] OR “pf 4522625”[Text Word] OR pf4522625[Text Word] OR preflucel[Text Word] OR prepandrix[Text Word] OR previgrip[Text Word] OR pumarix[Text Word] OR sandovac[Text Word] OR serinflu[Text Word] OR “skf 106160”[Text Word] OR supemtek[Text Word] OR trivalent[Text Word] OR ultragrivac[Text Word] OR vacciflu[Text Word] OR “vax 102”[Text Word] OR vax102[Text Word] OR vaxigrip[Text Word] OR vaxigriptetra[Text Word] OR vepacel[Text Word] OR viroflu[Text Word] OR “x-flu”[Text Word] OR xanaflu[Text Word]	
#10	"Measles-Mumps-Rubella Vaccine"[Mesh] OR immravax[Text Word] OR “m m rvaxpro”[Text Word] OR “m-m-r ii”[Text Word] OR “m-m-r vax”[Text Word] OR “m-m-rvaxpro”[Text Word] OR “m.m.r. ii”[Text Word] OR “mm rvaxpro”[Text Word] OR mmr[Text Word] OR mmrvaxpro[Text Word] OR morupar[Text Word] OR “mumeru vax”[Text Word] OR pluserix[Text Word] OR priorix[Text Word] OR “r.o.r. vax”[Text Word] OR “tri-kovax”[Text Word] OR trimovax[Text Word] OR triviraten[Text Word] OR trivirix[Text Word] OR virivac[Text Word]	11,231
#11	"Meningococcal Vaccines"[Mesh] OR “acwy vax”[Text Word] OR bexsero[Text Word] OR “men quad tt”[Text Word] OR menactra[Text Word] OR menacwy[Text Word] OR menafrivac[Text Word] OR menbvac[Text Word] OR mencevax[Text Word] OR meningitec[Text Word] OR meninvact[Text Word] OR menjugate[Text Word] OR menomune[Text Word] OR menpovax[Text Word] OR menquadfi[Text Word] OR menveo[Text Word] OR menzb[Text Word] OR “neisvac c”[Text Word] OR nimenrix[Text Word] OR nonamen[Text Word] OR “tetramen-t”[Text Word] OR trumenba[Text Word] OR “va mengoc bc”[Text Word] OR “va-mengoc-bc”[Text Word]	4,015
#12	"Pneumococcal Vaccines"[Mesh] OR moniarix[Text Word] OR “pcv 13”[Text Word] OR pcv13[Text Word] OR “phid cv”[Text Word] OR “pneu immune”[Text Word] OR “pneumo 23”[Text Word] OR “PPV23”[Text Word] OR	9,282

Search number	Search terms	Hits
	pneumopur[Text Word] OR pneumovax[Text Word] OR “pnu immune”[Text Word] OR “pnu imune”[Text Word] OR “pnu-imune 23”[Text Word] OR prevenar*[Text Word] OR prevnar[Text Word] OR streptopur[Text Word] OR streptorix[Text Word]	
#13	"Diphtheria-Tetanus Vaccine"[Mesh] OR “d t vax”[Text Word] OR “dif tet all”[Text Word] OR diftavax[Text Word] OR diftetall[Text Word] OR ditanrix[Text Word] OR ditebooster[Text Word] OR “dt reduct”[Text Word] OR “imovax d t”[Text Word] OR “td immun”[Text Word] OR “td pur”[Text Word] OR “td rix”[Text Word] OR tedivax[Text Word] OR "Diphtheria-Tetanus-Pertussis Vaccine"[Mesh] OR “acel immune”[Text Word] OR “acel imune”[Text Word] OR adacel[Text Word] OR “adsorbed dt coq”[Text Word] OR “anatoxal di te per berna”[Text Word] OR “booster tdap”[Text Word] OR boostertdap[Text Word] OR boostrix[Text Word] OR certiva[Text Word] OR covaxis[Text Word] OR “d.t. coq”[Text Word] OR daptacel[Text Word] OR “dif per tet all”[Text Word] OR “diphtheria plus pertussis plus tetanus”[Text Word] OR “diphtheria tetanus pertussis trivaccine”[Text Word] OR “diteki booster”[Text Word] OR ditekibooster[Text Word] OR dpt[Text Word] OR dtap*[Text Word] OR DTP[Text Word] OR Infanrix*[Text Word] OR neodiftepertus[Text Word] OR “p.d.t. vax purified”[Text Word] OR pertugen[Text Word] OR “tdap-immun”[Text Word] OR tdapbooster[Text Word] OR “tri immunol”[Text Word] OR triacelluvax[Text Word] OR triaxis[Text Word] OR tribaccine[Text Word] OR tripacel[Text Word] OR tripedia[Text Word] OR tripvac[Text Word] OR tritanrix[Text Word] OR trivax[Text Word]	7,727
#14	"Chickenpox Vaccine"[Mesh] OR okavax[Text Word] OR suduvax[Text Word] OR “v-z vax”[Text Word] OR “vaccin varilrix”[Text Word] OR varipox[Text Word] OR varivax[Text Word]	3,199
#15	"Herpes Zoster Vaccine"[Mesh] OR shingrix[Text Word] OR varilrix[Text Word] OR zostavax[Text Word]	1,166
#16	#4 OR #5 OR #6 OR #7 OR #8 OR #9 OR #10 OR #11 OR #12 OR #13 OR #14 OR #15	546,525
**Outcomes**		
#17	"Social Determinants of Health"[Mesh] OR “social determinant*”[Text Word] OR “social determining factor”[Text Word] OR “social health determinant”[Text Word] OR “social factors determining health”[Text Word] OR "Socioeconomic Factors"[Mesh] OR “economic value of life”[Text Word] OR “indigent health care”[Text Word] OR “medical indigency”[Text Word] OR “social economic*”[Text Word] OR “socio economic*”[Text Word] OR socioeconomic*[Text Word] OR "Health Status Disparities"[Mesh] OR "Health Inequities"[Mesh] OR “health disparit*”[Text Word] OR “health inequalit*”[Text Word] OR “health inequit*”[Text Word] OR “health status disparity”[Text Word] OR “health status disparities”[Text Word] OR “health status inequality”[Text Word] OR “health status inequalities”[Text Word] OR “health status inequity”[Text Word] OR “health status inequities”[Text Word] OR "Poverty"[Mesh] OR poverty[Text Word] OR indigence[Text Word] OR indigency[Text Word] OR impoverished[Text Word] OR “low income”[Text Word] OR “underserved population*”[Text Word] OR “underserved communit*”[Text Word] OR barriers[Text Word]	805,164

Search number	Search terms	Hits
**Geographic limitation**		
#18	"United States"[Mesh] OR "United States"[Title/Abstract] OR "USA"[Title/Abstract] OR "U.S.A."[Title/Abstract] OR "U.S."[Title/Abstract] OR "United States"[Affiliation] OR "USA"[Affiliation] OR "U.S.A."[Affiliation] OR "U.S."[Affiliation] OR "US"[Affiliation] OR Alabama[Affiliation] OR Alaska[Affiliation] OR Arizona[Affiliation] OR Arkansas[Affiliation] OR California[Affiliation] OR Colorado[Affiliation] OR Connecticut[Affiliation] OR Delaware[Affiliation] OR Florida[Affiliation] OR Georgia[Affiliation] OR Hawaii[Affiliation] OR Idaho[Affiliation] OR Illinois[Affiliation] OR Indiana[Affiliation] OR Iowa[Affiliation] OR Kansas[Affiliation] OR Kentucky[Affiliation] OR Louisiana[Affiliation] OR Maine[Affiliation] OR Maryland[Affiliation] OR Massachusetts[Affiliation] OR Michigan[Affiliation] OR Minnesota[Affiliation] OR Mississippi[Affiliation] OR Missouri[Affiliation] OR Montana[Affiliation] OR Nebraska[Affiliation] OR Nevada[Affiliation] OR “New Hampshire”[Affiliation] OR “New Jersey”[Affiliation] OR “New Mexico”[Affiliation] OR “New York”[Affiliation] OR “North Carolina”[Affiliation] OR “North Dakota”[Affiliation] OR Ohio[Affiliation] OR Oklahoma[Affiliation] OR Oregon[Affiliation] OR Pennsylvania[Affiliation] OR “Rhode Island”[Affiliation] OR “South Carolina”[Affiliation] OR “South Dakota”[Affiliation] OR Tennessee[Affiliation] OR Texas[Affiliation] OR Utah[Affiliation] OR Vermont[Affiliation] OR Virginia[Affiliation] OR Washington[Affiliation] OR “West Virginia”[Affiliation] OR Wisconsin[Affiliation] OR Wyoming[Affiliation] OR “Puerto Rico”[Affiliation] OR Alabama[Text Word] OR Alaska[Text Word] OR Arizona[Text Word] OR Arkansas[Text Word] OR California[Text Word] OR Colorado[Text Word] OR Connecticut[Text Word] OR Delaware[Text Word] OR Florida[Text Word] OR Georgia[Text Word] OR Hawaii[Text Word] OR Idaho[Text Word] OR Illinois[Text Word] OR Indiana[Text Word] OR Iowa[Text Word] OR Kansas[Text Word] OR Kentucky[Text Word] OR Louisiana[Text Word] OR Maine[Text Word] OR Maryland[Text Word] OR Massachusetts[Text Word] OR Michigan[Text Word] OR Minnesota[Text Word] OR Mississippi[Text Word] OR Missouri[Text Word] OR Montana[Text Word] OR Nebraska[Text Word] OR Nevada[Text Word] OR “New Hampshire”[Text Word] OR “New Jersey”[Text Word] OR “New Mexico”[Text Word] OR “New York”[Text Word] OR “North Carolina”[Text Word] OR “North Dakota”[Text Word] OR Ohio[Text Word] OR Oklahoma[Text Word] OR Oregon[Text Word] OR Pennsylvania[Text Word] OR “Rhode Island”[Text Word] OR “South Carolina”[Text Word] OR “South Dakota”[Text Word] OR Tennessee[Text Word] OR Texas[Text Word] OR Utah[Text Word] OR Vermont[Text Word] OR Virginia[Text Word] OR Washington[Text Word] OR “West Virginia”[Text Word] OR Wisconsin[Text Word] OR Wyoming[Text Word] OR “Puerto Rico”[Text Word]	7,879,208
**Exclusion terms**		
#19	“Animals”[MeSH] NOT “Humans”[MeSH]	5,029,737
#20	"Comment"[Publication Type] OR "Letter"[Publication Type] OR "Editorial"[Publication Type] OR "Case Reports"[Publication Type] OR "case study"[Title] OR "case studies"[Title] OR "case report"[Title] OR "case reports"[Title] OR "case series"[Title]	4,240,496

Search number	Search terms	Hits
**Total**		
#21	#3 AND #16 AND #17 AND #18	136
#22	#21 NOT (#19 OR #20)	134
#23	(#22) AND (("2005/01/01"[Date - Publication] : "3000"[Date - Publication]))	**101**

Table A3. Cochrane Search Strategy for the Systematic Literature Review on Factors Affecting the Uptake and Provision of Vaccines (Search Conducted 1 August 2022)

Search number	Search terms	Hits
**Population**		
#1	[mh Adult] OR adult* OR (grown NEXT up*):ti,ab,kw OR grownup* OR [mh ^Institutionalization] OR [mh “middle aged”] OR (middle NEXT age*):ti,ab,kw OR [mh "Young Adult"] OR [mh Aged] OR aged OR elderl* OR senior* OR senium OR [mh "Frail Elderly"] OR “very elderly” OR “80 and over” OR centenarian* OR nonagenarian* OR octogenarian* OR “very old” OR pensioner*	954,955
#2	[mh medicaid] OR Medicaid OR “Publicly Insured”	1,761
#3	#1 AND #2	1,048
**Intervention**		
#4	[mh Immunization] OR immunization* OR immunisation OR (immunostimulation NEAR/1 therap*):ti,ab,kw OR reimmunzation* OR reimmunization* OR [mh "Biolistics"] OR [mh "Mass Vaccination"] OR [mh "Immunization, Secondary"] OR [mh "Vaccination Coverage"] OR [mh Vaccination] OR vaccination* OR vaccine* OR vaccinotherap*	31,096
#5	“act hib” OR acthib OR “haemophilus b conjugate” OR hbpv OR “hib ompc” OR “hib vax” OR hiberix OR hibest OR hibtiter OR omnihib OR “pedvax hib” OR pedvaxhib OR prohibit OR “vaxem hib”	364
#6	[mh "Hepatitis A Vaccines"] OR avaxim OR epaxal OR havpur OR havrix OR “mevac a” OR nothav OR vaqta	355
#7	[mh "Hepatitis B Vaccines"] OR fendrix OR “gen h b vax” OR “gen hb vax” OR “h b vax” OR “hb vax” OR “hepaccine b” OR hepagene OR hepatavax OR “hepavaxx B” OR heplisav OR heprecomb OR “heptavax b” OR hepuman OR “hevac b” OR supervax OR “tgp 943” OR theradigm OR “v 270” OR v270	1,014
#8	[mh "Papillomavirus Vaccines"] OR ceravix OR cervarix OR gardasil OR silgard	611
#9	[mh "Influenza Vaccines"] OR “a rix” OR adjupanrix OR admune OR aflunov OR afluria OR aggripal OR agriflu OR agrippal OR “alfa rix” OR “alfa rix tetra” OR alorbat OR “alpha rix” OR “alpha rix tetra” OR anflu OR arepanrix OR batrevac OR begripal OR begrivac OR “bvx m001” OR celtura OR celvapan OR chiroflu OR chiromas OR daronrix OR enzira OR “flu immune” OR “flu imune” OR “flu vac” OR fluad OR fluarix* OR fluax OR flublok OR flucelvax OR fluenz OR flugen* OR fluinsure OR flulaval OR flumist OR fluogen OR flushield OR flustat OR fluvaccinol OR fluvarix OR fluvax OR fluviral OR fluvirin* OR fluviron OR fluzone OR focetria OR foclivia OR gammaflu OR grippovac OR humenza OR idflu OR immugrip OR imuvac OR inflexal OR influject OR influpozzi OR influsplit OR influvac OR intanza OR “inviron ol” OR invivac OR iradogen OR istivac OR mastaflu OR “medi 3314” OR medi3314 OR “mfv ject” OR “multimeric 001” OR munevan OR mutagrip OR nasovax OR nivgrip OR optaflu OR pandemrix OR panenza OR panvax OR “pf 4522625” OR pf4522625 OR preflucel OR prepandrix OR previgrip OR pumarix OR sandovac OR serinflu OR “skf 106160” OR supemtek OR trivalent OR	2,895

Search number	Search terms	Hits
	ultragrivac OR vacciflu OR “vax 102” OR vax102 OR vaxigrip OR vaxigriptetra OR vepacel OR viroflu OR “x-flu” OR xanaflu	
#10	[mh "Measles-Mumps-Rubella Vaccine"] OR immravax OR “m m rvaxpro” OR “m m r ii” OR “m m r vax” OR “m m rvaxpro” OR “m.m.r. ii” OR “mm rvaxpro” OR mmr OR mmrvaxpro OR morupar OR “mumeru vax” OR pluserix OR priorix OR “r.o.r. vax” OR “tri kovax” OR trimovax OR triviraten OR trivirix OR virivac	1,253
#11	[mh "Meningococcal Vaccines"] OR “acwy vax” OR bexsero OR “men quad tt” OR menactra OR menacwy OR menafrivac OR menbvac OR mencevax OR meningitec OR meninvact OR menjugate OR menomune OR menpovax OR menquadfi OR menveo OR menzb OR “neisvac c” OR nimenrix OR nonamen OR “tetramen t” OR trumenba OR “va mengoc bc”	728
#12	[mh "Pneumococcal Vaccines"] OR moniarix OR “pcv 13” OR pcv13 OR “phid cv” OR “pneu immune” OR “pneumo 23” OR “PPV23” OR pneumopur OR pneumovax OR “pnu immune” OR “pnu imune” OR “pnu imune 23” OR prevenar* OR prevnar OR streptopur OR streptorix	1,420
#13	[mh "Diphtheria-Tetanus Vaccine"] OR “d t vax” OR “dif tet all” OR diftavax OR diftetall OR ditanrix OR ditebooster OR “dt reduct” OR “imovax d t” OR “td immun” OR “td pur” OR “td rix” OR tedivax OR [mh "Diphtheria-Tetanus-Pertussis Vaccine"] OR “acel immune” OR “acel imune” OR adacel OR “adsorbed dt coq” OR “anatoxal di te per berna” OR (booster NEAR/1 tdap):ti,ab,kw OR boostertdap OR boostrix OR certiva OR covaxis OR “d.t. coq” OR daptacel OR “dif per tet all” OR “diphtheria plus pertussis plus tetanus” OR “diphtheria tetanus pertussis trivaccine” OR “diteki booster” OR ditekibooster OR dpt OR dtap* OR DTP OR Infanrix* OR neodiftepertus OR “p.d.t. vax purified” OR pertugen OR “tdap immun” OR tdapbooster OR “tri immunol” OR triacelluvax OR triaxis OR tribaccine OR tripacel OR tripedia OR tripvac OR tritanrix OR trivax	1,863
#14	[mh "Chickenpox Vaccine"] OR okavax OR suduvax OR “v z vax” OR “vaccin varilrix” OR varipox OR varivax	254
#15	[mh "Herpes Zoster Vaccine"] OR shingrix OR varilrix OR zostavax	183
#16	#4 OR #5 OR #6 OR #7 OR #8 OR #9 OR #10 OR #11 OR #12 OR #13 OR #14 OR #15	32,617
**Outcomes**		
#17	[mh "Social Determinants of Health"] OR (social NEXT determinant*):ti,ab,kw OR “social determining factor” OR “social health determinant” OR “social factors determining health” OR [mh "Socioeconomic Factors"] OR “economic value of life” OR “indigent health care” OR “medical indigency” OR (social NEXT economic*):ti,ab,kw OR (socio NEXT economic*):ti,ab,kw OR socioeconomic* OR [mh "Health Status Disparities"] OR [mh "Health Inequities"] OR (health NEXT disparit*):ti,ab,kw OR (health NEXT inequalit*):ti,ab,kw OR (health NEXT inequit*):ti,ab,kw OR “health status disparity” OR “health status disparities” OR “health status inequality” OR “health status inequalities” OR “health status inequity” OR “health status inequities” OR [mh Poverty] OR poverty OR indigence OR indigency OR	37,713

Search number	Search terms	Hits
	impoverished OR “low income” OR (underserved NEXT population*):ti,ab,kw OR (underserved NEXT communit*):ti,ab,kw OR barriers	
**Geographic limitation**		
#18	[mh “united states”] OR “united states” OR “usa” OR “u.s.a.” OR (“u.s.” NOT “Non-U.S.”) OR alabama OR alaska OR arizona OR arkansas OR california OR colorado OR connecticut OR delaware OR florida OR georgia OR hawaii OR idaho OR illinois OR indiana OR iowa OR kansas OR kentucky OR louisiana OR maine OR maryland OR massachusetts OR michigan OR minnesota OR mississippi OR missouri OR montana OR nebraska OR nevada OR “new hampshire” OR “new jersey” OR “new mexico” OR “new york” OR “north carolina” OR “north dakota” OR ohio OR oklahoma OR oregon OR pennsylvania OR “rhode island” OR “south carolina” OR “south dakota” OR tennessee OR texas OR utah OR vermont OR virginia OR washington OR “west virginia” OR wisconsin OR wyoming OR “puerto rico”	346,215
**Exclusion terms**		
#19	[mh Animals] NOT [mh Humans]	22
#20	("Comment" OR "Letter" OR "Editorial" OR "Case Reports" OR “conference abstract”):pt OR ("case study" OR "case studies" OR "case report" OR "case reports" OR "case series"):ti	214,083
**Total**		
#21	#3 AND #16 AND #17 AND #18	42
#22	#21 NOT (#19 OR #20)	40
#23	#21 NOT (#19 OR #20) with Cochrane Library publication date from Jan 2005 to present, in Cochrane Reviews	22
#24	#21 NOT (#19 OR #20) with Publication Year from 2005 to 2022, with Cochrane Library publication date from Jan 2005 to present, in Trials	13
#25	#23 OR #24	35

Table A4. CRD (DARE) Search Strategy for the Systematic Literature Review on Factors Affecting the Uptake and Provision of Vaccines (Search Conducted 1 August 2022)

Search number	Search terms	Hits
**Population**		
#1	MeSH DESCRIPTOR adult EXPLODE ALL TREES	17,789
#2	MeSH DESCRIPTOR Institutionalization	31
#3	MeSH DESCRIPTOR middle aged EXPLODE ALL TREES	11,186
#4	MeSH DESCRIPTOR Young Adult EXPLODE ALL TREES	1,941
#5	MeSH DESCRIPTOR Aged EXPLODE ALL TREES	9,687
#6	MeSH DESCRIPTOR Frail Elderly EXPLODE ALL TREES	84
#7	(adult* OR grown up* OR grownup* OR middle age* OR aged OR elderl* OR senior* OR senium OR very elderly OR 80 and over OR centenarian* OR nonagenarian* OR octogenarian* OR very old OR pensioner*)	10,528
#8	#1 OR #2 OR #3 OR #4 OR #5 OR #6 OR #7	21,387
#9	MeSH DESCRIPTOR medicaid EXPLODE ALL TREES	74
#10	(Medicaid OR Publicly Insured)	308
#11	#9 OR #10	308
#12	#8 AND #11	180
**Intervention**		
#13	MeSH DESCRIPTOR Immunization EXPLODE ALL TREES	679
#14	MeSH DESCRIPTOR Biolistics EXPLODE ALL TREES	0
#15	MeSH DESCRIPTOR Mass Vaccination EXPLODE ALL TREES	59
#16	MeSH DESCRIPTOR Immunization, Secondary EXPLODE ALL TREES	33
#17	MeSH DESCRIPTOR Vaccination Coverage EXPLODE ALL TREES	0
#18	MeSH DESCRIPTOR Vaccination EXPLODE ALL TREES	527
#19	(immunization* OR immunisation OR immunostimulation therap* OR reimmunzation* OR reimmunization* OR vaccination* OR vaccine* OR vaccinotherap*)	1,667
#20	(act hib OR acthib OR haemophilus b conjugate OR hbpv OR hib ompc OR hib vax OR hiberix OR hibest OR hibtiter OR omnihib OR pedvax hib OR pedvaxhib OR prohibit OR vaxem hib)	8
#21	MeSH DESCRIPTOR Hepatitis A Vaccines EXPLODE ALL TREES	42
#22	(avaxim OR epaxal OR havpur OR havrix OR mevac a”OR nothav OR vaqta)	8
#23	MeSH DESCRIPTOR Hepatitis B Vaccines EXPLODE ALL TREES	77
#24	(fendrix OR gen h b vax OR gen hb vax OR h b vax OR hb vax OR hepaccine b OR hepagene OR hepatavax OR hepavaxx B OR heplisav OR heprecomb OR heptavax b OR hepuman OR hevac b OR supervax OR tgp 943 OR theradigm OR v 270 OR v270)	4
#25	MeSH DESCRIPTOR Papillomavirus Vaccines EXPLODE ALL TREES	152

Search number	Search terms	Hits
#26	(ceravix OR cervarix OR gardasil OR silgard)	6
#27	MeSH DESCRIPTOR Influenza Vaccines EXPLODE ALL TREES	201
#28	(a rix OR adjupanrix OR admune OR aflunov OR afluria OR aggripal OR agriflu OR agrippal OR alfa rix OR alfa rix tetra OR alorbat OR alpha rix OR alpha rix tetra OR anflu OR arepanrix OR batrevac OR begripal OR begrivac OR bvx m001 OR celtura OR celvapan OR chiroflu OR chiromas OR daronrix OR enzira OR flu immune OR flu imune OR flu vac OR fluad OR fluarix* OR fluax OR flublok OR flucelvax OR fluenz OR flugen* OR fluinsure OR flulaval OR flumist OR fluogen OR flushield OR flustat OR fluvaccinol OR fluvarix OR fluvax OR fluviral OR fluvirin* OR fluviron OR fluzone OR focetria OR foclivia OR gammaflu OR grippovac OR humenza OR idflu OR immugrip OR imuvac OR inflexal OR influject OR influpozzi OR influsplit OR influvac OR intanza OR inviron ol OR invivac OR iradogen OR istivac OR mastaflu OR medi 3314 OR medi3314 OR mfv ject OR multimeric 001 OR munevan OR mutagrip OR nasovax OR nivgrip OR optaflu OR pandemrix OR panenza OR panvax OR pf 4522625 OR pf4522625 OR preflucel OR prepandrix OR previgrip OR pumarix OR sandovac OR serinflu OR skf 106160 OR supemtek OR trivalent OR ultragrivac OR vacciflu OR vax 102 OR vax102 OR vaxigrip OR vaxigriptetra OR vepacel OR viroflu OR x flu OR xanaflu)	36
#29	MeSH DESCRIPTOR Measles-Mumps-Rubella Vaccine EXPLODE ALL TREES	20
#30	(immravax OR m m rvaxpro OR m m r ii OR m m r vax OR m m rvaxpro OR m.m.r. ii OR mm rvaxpro OR mmr OR mmrvaxpro OR morupar OR mumeru vax OR pluserix OR priorix OR r.o.r. vax OR tri kovax OR trimovax OR triviraten OR trivirix OR virivac)	43
#31	MeSH DESCRIPTOR Meningococcal Vaccines EXPLODE ALL TREES	28
#32	(acwy vax OR bexsero OR men quad tt OR menactra OR menacwy OR menafrivac OR menbvac OR mencevax OR meningitec OR meninvact OR menjugate OR menomune OR menpovax OR menquadfi OR menveo OR menzb OR neisvac c OR nimenrix OR nonamen OR tetramen t OR trumenba OR va mengoc bc)	2
#33	MeSH DESCRIPTOR Pneumococcal Vaccines EXPLODE ALL TREES	168
#34	(moniarix OR pcv 13 OR pcv13 OR phid cv OR pneu immune OR pneumo 23 OR PPV23 OR pneumopur OR pneumovax OR pnu immune OR pnu imune OR pnu imune 23 OR prevenar* OR prevnar OR streptopur OR streptorix)	33
#35	MeSH DESCRIPTOR Diphtheria-Tetanus Vaccine EXPLODE ALL TREES	2
#36	MeSH DESCRIPTOR Diphtheria-Tetanus-Pertussis Vaccine EXPLODE ALL TREES	17
#37	(d t vax OR dif tet all OR diftavax OR diftetall OR ditanrix OR ditebooster OR dt reduct OR imovax d t OR td immun OR td pur OR td rix OR tedivax OR acel immune OR acel imune OR adacel OR adsorbed dt coq OR anatoxal di te per berna OR booster tdap OR boostertdap OR boostrix OR certiva OR covaxis OR d.t. coq OR daptacel OR dif per tet all OR diphtheria plus pertussis plus tetanus OR diphtheria tetanus pertussis trivaccine OR diteki booster OR ditekibooster OR dpt OR dtap* OR DTP OR Infanrix* OR neodiftepertus OR	28

Search number	Search terms	Hits
	p.d.t. vax purified OR pertugen OR tdap immun OR tdapbooster OR tri immunol OR triacelluvax OR triaxis OR tribaccine OR tripacel OR tripedia OR tripvac OR tritanrix OR trivax)	
#38	MeSH DESCRIPTOR Chickenpox Vaccine EXPLODE ALL TREES	54
#39	(okavax OR suduvax OR v z vax OR vaccin varilrix OR varipox OR varivax)	2
#40	MeSH DESCRIPTOR Herpes Zoster Vaccine EXPLODE ALL TREES	15
#41	(shingrix OR varilrix OR zostavax)	5
#42	#13 OR #14 OR #15 OR #16 OR #17 OR #18 OR #19 OR #20 OR #21 OR #22 OR #23 OR #24 OR #25 OR #26 OR #27 OR #28 OR #29 OR #30 OR #31 OR #32 OR #33 OR #34 OR #35 OR #36 OR #37 OR #38 OR #39 OR #40 OR #41	32,617
**Outcomes**		
#43	MeSH DESCRIPTOR Social Determinants of Health EXPLODE ALL TREES	0
#44	MeSH DESCRIPTOR Socioeconomic Factors EXPLODE ALL TREES	808
#45	MeSH DESCRIPTOR Health Status Disparities EXPLODE ALL TREES	24
#46	MeSH DESCRIPTOR Health Inequities EXPLODE ALL TREES	0
#47	MeSH DESCRIPTOR Poverty EXPLODE ALL TREES	121
#48	(social determinant* OR social determining factor OR social health determinant OR social factors determining health OR economic value of life OR indigent health care OR medical indigency OR social economic* OR socio economic* OR socioeconomic* OR health disparit* OR health inequalit* OR health inequit* OR health status disparity OR health status disparities OR health status inequality OR health status inequalities OR health status inequity OR health status inequities OR poverty OR indigence OR indigency OR impoverished OR low income OR underserved population* OR underserved communit* OR barriers)	1,337
#49	#43 OR #44 OR #45 OR #46 OR #47 OR #48	1,788
**Geographic limitation**		
#50	MeSH DESCRIPTOR united states EXPLODE ALL TREES	3,279
#51	(usa OR u.s.a. OR u.s. OR alabama OR alaska OR arizona OR arkansas OR california OR colorado OR connecticut OR delaware OR florida OR georgia OR hawaii OR idaho OR illinois OR indiana OR iowa OR kansas OR kentucky OR louisiana OR maine OR maryland OR massachusetts OR michigan OR minnesota OR mississippi OR missouri OR montana OR nebraska OR nevada OR new hampshire OR new jersey OR new mexico OR new york OR north carolina OR north dakota OR ohio OR oklahoma OR oregon OR pennsylvania OR rhode island OR south carolina OR south dakota OR tennessee OR texas OR utah OR vermont OR virginia OR washington OR west virginia OR wisconsin OR wyoming OR puerto rico)	346,215
#52	#50 OR #51	9,331
**Total**		
#53	#12 AND #42 AND #49 AND #52	1

Search number	Search terms	Hits
#54	(#12 AND #42 AND #49) IN DARE FROM 2005 TO 2022	0

Table A5. Study Characteristics – Patients

Author (date), title, US state	Vaccine type/s	Study details[a]	Inclusion/ exclusion criteria	Patient characteristics	Provider characteristics	Outcomes available
**Author:** Kim et al. (2021)<br>**Title:** *Examining the effects of the Medicaid expansion on health outcomes*<br>**State:** 17 Medicaid expansion states and 19 non-expansion states	Influenza	**Study type**: Cross-sectional study<br>**Data source:** The BRFSS<br>**Objective:**<br>To examine the impact of Medicaid expansion on healthcare resource utilization, health outcomes and access to healthcare<br>**Data vintage:**<br>▪ 2012-2013 (before Medicaid expansion)<br>▪ 2015-2016 (after Medicaid expansion)	14 states and the District of Columbia were excluded due to early or late ACA Medicaid expansions or the presence of a pre-ACA Medicaid waiver covering childless adults up to 100% of the federal poverty level. 2014 data were excluded due to Medicaid expansion enrollments occurring that year.	NR	NA	Patient general barriers
**Author:** Hatch et al. (2021)<br>**Title:** *Impacts of the Affordable Care Act on*	HPV, influenza	**Study type**: Cross-sectional study	Community health centers were included if they had live EHR systems as of 1	**Overall N (expansion states) = 392,703**<br>▪ Mean (SD) age: 37.7 (14.9)	NA	Patient focused barriers

Author (date), title, US state	Vaccine type/s	Study details[a]	Inclusion/ exclusion criteria	Patient characteristics	Provider characteristics	Outcomes available
receipt of women's preventive services in community health centers in Medicaid expansion and non-expansion states<br>**State:** 10 Medicaid expansion states and 4 non-expansion states		**Data source:** EHR data from ADVANCE<br>**Objective:** To assess whether ACA implementation and Medicaid expansion were followed by greater receipt of recommended preventive services among women and girls in a large network of community health centers<br>**Data vintage:**<br>▪ 2012-2013 (before Medicaid expansion)<br>▪ 2014-2015 (after Medicaid expansion)	January 2012, provided primary and preventive care services to women, and were located in states that either expanded Medicaid under the ACA on 1 January 2014, or did not expand by the study period's end (31 December 2015). Patients included were nonpregnant females aged 11-65 who were eligible (or due) for at least 1 focal preventive service, and had at least 1 ambulatory visit during the 2-year time period of interest. Women with any indication of pregnancy	▪ Mean (SD) Charlson score: 1.3 (2.0)<br>▪ Medicaid: 186,812 (47.6%)		

Author (date), title, US state	Vaccine type/s	Study details[a]	Inclusion/ exclusion criteria	Patient characteristics	Provider characteristics	Outcomes available
			during the study were excluded.			
**Author:** Osazuwa-Peters et al. (2020)<br>**Title:** *The Affordable Care Act and rate of human papillomavirus (HPV) vaccine uptake in the United States*<br>**State:** Nationwide	HPV	**Study type**: Cross-sectional study<br>**Data source:** NHIS<br>**Objective:** Evaluate the association between the ACA and know HPV-vaccination-enabling factors<br>**Data vintage:**<br>▪ 2011-2013 (pre-ACA)<br>▪ 2014-2017 (post-ACA)	Individuals aged 18-26 years were included.	▪ **Overall N (pre-ACA) = 13,494**<br>▪ **Overall N (post-ACA) = 15,722**	NR	Patient general barriers
**Author:** Aris et al. (2020)<br>**Title:** *The adult vaccination landscape in the United States during the Affordable Care Act era: results from a large*	Multiple: Tdap, HZ, pneumococcal	**Study type**: Retrospective database analysis<br>**Data source:** Truven Health MarketScan CCAE, MS, and multi-state Medicaid databases.	**Objective 1:**<br>Individuals aged ≥ 19 years by 31 December 2016 were included in the analysis.<br>**Objective 2,3,4, 5 (influenza):** Continuously enrolled Sep-Feb in	**Eligible vaccine cohorts**<br>**Influenza:**<br>▪ N **=** 98,060,151<br>▪ Female = 52,028,227 (53.06%)<br>**HZ:**<br>▪ N = 1,146,297<br>▪ Female: 619,304 (54.03%)	NA	Patient focused barriers;<br>1 mention of improving support to providers

Author (date), title, US state	Vaccine type/s	Study details[a]	Inclusion/ exclusion criteria	Patient characteristics	Provider characteristics	Outcomes available
retrospective database analysis<br>**State:** Nationwide		**Objective:**<br>The objectives of the study were to:<br>1) Examine the characteristics of visits where adults received influenza, Tdap, HZ, and pneumococcal vaccination.<br>2) Describe well-visit utilization (i.e., preventive care or vaccine-only visits) around the times and ages of recommended influenza, HZ, and pneumococcal vaccinations.<br>3) Evaluate potential missed opportunities for influenza, HZ, and pneumococcal vaccination.	at least 1 flu season of 2011-12, 2012-13, 2013-14, 2014-15, or 2015-16 and had at least 1 well-visit.<br>**Objective 2,3,4, 5 (HZ):**<br>Born in 1951-54 and had 36 months of continuous enrollment starting 1 Jan in the year the individual reached age 60 years and had at least 1 well-visit<br>**Objective 2,3,4, 5 (Pneumococcal):**<br>Born in 1946-49 and had 36 months of continuous enrollment starting 1 Jan in the year the individual reached age 65 years and had at least 1 well-visit.	**Pneumococcal:**<br>▪ N = 412,500<br>▪ Female: 212,513 (51.52%)		

Author (date), title, US state	Vaccine type/s	Study details[a]	Inclusion/ exclusion criteria	Patient characteristics	Provider characteristics	Outcomes available
		4) Identify factors associated with influenza, HZ, and pneumococcal vaccination.<br>5) Identify factors associated with missed opportunities for influenza, HZ, and pneumococcal vaccination.<br>**Data vintage:** January 2011 through December 2016				
**Author:** Hurley et al. (2019)<br>**Title:** *Randomized controlled trial of centralized vaccine reminder/recall to improve adult vaccination rates in an accountable care organization setting*	Influenza, pneumococcal, Td/Tdap	**Study type**: Randomized controlled trial<br>**Data source:** CIIS<br>**Objective:** Assess effectiveness of using CIIS to send out vaccine reminder/recalls centrally vs. usual care and practice staff's perception	Practices included were internal or family medicine practices, actively used the CIIS for adult vaccination data, and had at least 50 adults enrolled in the regional ACO. Patients included were aged	**Overall N (ages 19-64 intervention group) = 7,612**<br>▪ Female: 62%<br>▪ Median (IQR) age: 36 (26-50)<br>▪ Up to date on influenza at baseline: 0.1%<br>▪ Up to date on Tdap at baseline: 53%	6 practices included, including 2 FQHCs, 2 university clinics, and 2 private practices	Patient and provider general barriers

Author (date), title, US state	Vaccine type/s	Study details[a]	Inclusion/ exclusion criteria	Patient characteristics	Provider characteristics	Outcomes available
**State:** Colorado		of centralized reminder/recalls<br>**Trial time horizon:** 2016-2018	19 years and older as of 1 Sep 2016 and were deficient in at least 1 of the 3 vaccines being evaluated.	**Overall N (ages 65+ group) = 307**<br>Female: 64%<br>▪ Median (IQR) age: 71 (67-78)<br>▪ Up to date on influenza at baseline: 0.7%<br>▪ Up to date on Tdap at baseline: 48%<br>▪ Up to date on pneumococcal at baseline: 26%		
**Author:** Wheldon et al. (2021)<br>**Title:** *Decision support needs for shared clinical decision-making regarding HPV vaccination among adults 27-45 years of age*<br>**State:** National	HPV	**Study type**: Cross-sectional study<br>**Data source:** Qualtrics Online Panel<br>**Objective:** To identify HPV information needs and shared clinical decision-making preferences among adults 27-45 and describe differences in needs and preferences	Individuals between the ages of 27 and 45 years with no previous history of receipt of HPV vaccination. Additionally, African American and Hispanic populations were oversampled due to the lower prevalence of HPV vaccination among these ethnoracial groups compared	There were 702 survey respondents; of those, 470 (67%) were based in a Medicaid expansion state. Health insurance status for respondents were:<br>▪ Private: 387 (55.1%)<br>▪ Public: 212 (30.2%)<br>▪ Uninsured/don't know: 103 (14.7%)	NA	Patient barriers, with a focus on patient knowledge and information

Author (date), title, US state	Vaccine type/s	Study details[a]	Inclusion/ exclusion criteria	Patient characteristics	Provider characteristics	Outcomes available
		among underserved and vulnerable populations<br>**Data vintage:** April-May 2020	with non-Hispanic White populations.			
**Author:** Bloodworth et al. (2018)<br>**Title:** *Variation of preventive service utilization by state Medicaid coverage, cost-sharing, and Medicaid expansion status*<br>**State:** Michigan	Influenza	**Study type**: Data source analysis<br>**Data Source:** Kaiser Family Foundation and MEPS data<br>**Objective:** To examine the variation of preventive service utilization and disparities of preventive service utilization under various Medicaid policies<br>**Data vintage:** 2009-2014	All individuals in MEPS 2009-2014 who reported continuous Medicaid coverage over the past year and were eligible for at least 1 of the 3 preventive services analyzed.	Medicaid patients included in the analysis N = 15,610<br>**Sex**<br>Female: 64%<br>**Ethnicity**<br>White: 43.18%<br>Black: 23.58%<br>Asian/Hawaiian/Pacific Islander: 6.02%<br>Latino: 23.01%<br>Other: 4.21%	NR	Patient barriers and influenza vaccination utilization
**Author:** Stewart et al. (2014)<br>**Title:** *Vaccination benefits and cost-sharing policy for noninstitutionalize*	All adult vaccines	**Study type**: Document review and survey<br>**Data source:** Study survey of Medicaid	Medicaid administrators from 50 states and the District of Columbia were included in the	NA	NA	Patient focused barriers

Author (date), title, US state	Vaccine type/s	Study details[a]	Inclusion/ exclusion criteria	Patient characteristics	Provider characteristics	Outcomes available
d adult Medicaid enrollees in the United States **State:** Nationwide, but 9 states did not participate		administrators nationwide **Objective:** Examine state Medicaid programs' policies related to vaccine benefit coverage and copayments for adult enrollees **Data vintage:** 2012 (compared with 2003 results from previous study)	survey. Vaccination benefit and cost-sharing policies for noninstitutionalize d adult Medicaid enrollees were analyzed.			
**Author:** New et al. (2018) **Title:** *Barriers to receipt of prenatal tetanus toxoid, reduced diphtheria toxoid, and acellular pertussis vaccine among mothers of infants aged < 4 months with pertussis-California, 2016* **State:** California	Tdap	**Study type:** Interviews and supplemental questionnaire conducted as part of routine case investigations **Data source:** Case report form completed by local health department personnel; supplemental questionnaire using information	Mothers whose infants (aged < 4 months) developed pertussis in 2016, and their prenatal care providers	Overall, 66 mothers and their prenatal care providers completed the supplemental questionnaire during routine case investigations (out of 114 pertussis cases reported in 2016)	Prenatal care providers	Patient barriers, with a focus on vaccine opportunity, vaccine access, healthcare insurance, and personal reasons; provider barriers, with a focus on [illegible] and [illegible] issues preventing Tdap from being stocked onsite, [illegible] provider education

Author (date), title, US state	Vaccine type/s	Study details[a]	Inclusion/ exclusion criteria	Patient characteristics	Provider characteristics	Outcomes available
		collected during routine case investigation interviews; interviews with prenatal care providers; California Immunization Registry (mandated in late 2016 for pharmacists only)<br>**Objective:** To ascertain possible reasons for low Tdap coverage among pregnant women in California<br>**Data vintage:** 2016 (low-incidence pertussis year in California)				about Tdap vaccine recommendations
**Author:** Brown et al. (2018)<br>**Title:** *Evaluation of healthy fit: a community health*	Influenza, pneumonia, HPV	**Study type:** Longitudinal observational evaluation study	Uninsured and underinsured people, and Medicaid	In total, there were 514 participants; of these participants, 271 were recruited at the Mexican Consulate	NA	Patient barriers to redeem vaccine vouchers: lack of time and

Author (date), title, US state	Vaccine type/s	Study details[a]	Inclusion/ exclusion criteria	Patient characteristics	Provider characteristics	Outcomes available
worker model to address Hispanic health disparities<br>**State:** Texas		**Data source:** Follow-up telephone interviews<br>**Objective:** To identify the health needs of a predominantly Hispanic population living on the US–Mexico border; and to estimate participants responsiveness to recommendations and referrals provided by community health workers to address those needs<br>**Data vintage:** February 2015 through May 2016	beneficiaries aged 18 years or older.<br>Pregnant women were excluded because of the interest in tracking BMI changes in response to 1 of the interventions.	and 58 were recruited at a nonprofit community center<br>▪ Mean age (range): 45.9 years (18-94)<br>▪ Female: 81.9%<br>▪ Hispanic/Latino: 96.9%<br>▪ Type of health insurance:<br>– Private: 10.5%<br>– Medicare: 7.0%<br>– Medicaid: 3.1%<br>– Other: 1.6%<br>▪ No health insurance: 79.2%		indecision; these vouchers were provided in the context of 1,115 Medicaid waiver programs
**Author:** Stoecker et al. (2017)<br>**Title:** *The cost of cost-sharing: the impact of Medicaid*	Influenza	**Study type:** Secondary analysis of publicly available survey data using a	Adults aged 19-64 years regardless of income (respondents were	Imputed Medicaid population (pregnant women):<br>▪ 2003: 1 million<br>▪ 2012: 0.7 million	NA	Patient barriers, with a focus on financial incentives for Medicaid

Author (date), title, US state	Vaccine type/s	Study details[a]	Inclusion/ exclusion criteria	Patient characteristics	Provider characteristics	Outcomes available
benefit design on influenza vaccination uptake<br>**State:** Nationwide (49 states, excluding the District of Columbia and Florida)		differences-in-differences framework<br>**Data source:** Data on vaccine coverage were derived from 2 previous analyses of Medicaid benefit design (methods involved document review and survey); data on vaccination coverage was obtained from the BRFSS (telephone survey); Kaiser Family Foundation for the share of each state Medicaid population covered under fee-for-service Medicaid<br>**Objective:** To examine the effect of copayment charges and other	categorized as Medicaid enrolled if the midpoint of their reported household income range was below the state limit for Medicaid eligibility for the particular qualifying category, they reported having health insurance coverage, and they fell into 1 of the Medicaid eligibility categories included in this analysis); women who reported being pregnant at the time of the survey, and respondents of either sex who reported there were children living in the household.	Imputed Medicaid population (parents):<br>▪ 2003: 13.3 million<br>▪ 2012: 10.6 million<br>Percentage of respondents in states covering influenza vaccination (number of states):<br>▪ 2003: 85.4% (43)<br>▪ 2012: 93.7% (49)<br>Percentage of respondents in states prohibiting cost-sharing (number of states):<br>▪ 2003: 0% (0)<br>▪ 2012: 24.6% (20)		beneficiaries (i.e., coverage, prohibiting cost-sharing, and copayments)

Author (date), title, US state	Vaccine type/s	Study details[a]	Inclusion/ exclusion criteria	Patient characteristics	Provider characteristics	Outcomes available
		related elements of vaccination benefit design on influenza vaccination coverage among low-income adults; key variables related to Medicaid included:<br>1) Whether the state covered influenza vaccines for traditionally eligible Medicaid beneficiaries<br>2) Whether the state prohibited cost-sharing for vaccination services provided to adult Medicaid beneficiaries<br>3) Copayment amount charge<br>**Data vintage:** 2003 and 2012				

Author (date), title, US state	Vaccine type/s	Study details[a]	Inclusion/ exclusion criteria	Patient characteristics	Provider characteristics	Outcomes available
**Author:** Yue et al. (2018)<br>**Title:** *Racial/ethnic differential effects of Medicaid expansion on health care access*<br>**State:** nationwide	Influenza	**Study type:** Quasi-experimental study<br>**Data source:** the 2013 and 2015 BRFSS. The BRFSS consists of annual telephone surveys of noninstitutionalized adults ages 18 or older, collecting information on health status, access to care, health behaviors, and other demographic characteristics. The survey collected nationally representative samples using a stratified probability sampling design. The BRFSS dataset includes state identifiers, allowing for	Low-income, nonelderly adults were identified based on age, household income, and family size.	**Non-expansion states**<br>▪ Total observations: 18,408<br>▪ Age (SD): 44.55 (0.63) years<br>▪ Female: 12,357/18,408 (67.12%) (weighted %-60.75%)[b]<br>**Expansion states**<br>▪ Total observations: 16,946<br>▪ Age (SD): 43.66 (0.60) years<br>▪ Female: 11,085/16,946 (65.41%) (weighted %-59.45)[b]	NR	Impact of the ACA in Medicaid expansion states and the uptake of the influenza vaccine compared with non-expansion states, plus racial disparities

Author (date), title, US state	Vaccine type/s	Study details[a]	Inclusion/ exclusion criteria	Patient characteristics	Provider characteristics	Outcomes available
		distinction between expansion and non-expansion states. State Physicians Workforce Data Book and Bureau of Labor Statistics were also used.<br>**Objective:** To assess racial/ethnic differential impacts of the ACA's Medicaid expansion on low-income, nonelderly adults' access to primary care.<br>**Data vintage:** 2013-2015				
**Author:** Hawkins et al. (2021)<br>**Title:** *Associations between ACA-related policies and a clinical recommendation*	HPV	**Study type**: Retrospective claims database analysis<br>**Data source:** APCDs from New Hampshire (Comprehensive Health Care	The analytic sample included people aged 9-26 years residing in New Hampshire, Maine, and Massachusetts.	**Total N (received HPV vaccine) = 551,764**	NA	Patient general barriers

Author (date), title, US state	Vaccine type/s	Study details[a]	Inclusion/ exclusion criteria	Patient characteristics	Provider characteristics	Outcomes available
with HPV vaccine initiation<br>**State:** Maine, New Hampshire, Massachusetts		Information System), Maine (Maine Health Data Organization), and Massachusetts (Center for Health Information and Analysis)<br>**Objective:** Examine associations between the ACA, ACIP recommendation for males, and ACA-related health insurance reforms with HPV vaccine initiation rates by sex and test differences by health insurance type<br>**Data vintage:** 2009-2015				
**Author:** Hawkins et al. (2021)<br>**Title:** *Associations between ACA-related*	HPV	**Study type**: Retrospective claims database analysis	The analytic sample included people aged 9-26 years residing in New	**Total N (received HPV vaccine) = 551,764**	NA	Patient general barriers

Author (date), title, US state	Vaccine type/s	Study details[a]	Inclusion/ exclusion criteria	Patient characteristics	Provider characteristics	Outcomes available
policies and a clinical recommendation with HPV vaccine initiation **State:** Maine, New Hampshire, Massachusetts		**Data source:** APCDs from New Hampshire (Comprehensive Health Care Information System), Maine (Maine Health Data Organization), and Massachusetts (Center for Health Information and Analysis) **Objective:** Examine associations between the ACA, ACIP recommendation for males, and ACA-related health insurance reforms with HPV vaccine initiation rates by sex and test differences by health insurance type	Hampshire, Maine, and Massachusetts.			

Author (date), title, US state	Vaccine type/s	Study details[a]	Inclusion/ exclusion criteria	Patient characteristics	Provider characteristics	Outcomes available
		**Data vintage:** 2009-2015				
**Author:** Okoro et al. (2017)<br>**Title:** *Surveillance for health care access and health services use, adults aged 18-64 years-behavioral risk factor surveillance system, United States, 2014*<br>**States:** Nationwide (50 states and the District of Columbia)	Influenza	**Study type:** Survey<br>**Data source:** The BRFSS; an ongoing, state-based, landline- and cellular-phone based survey<br>**Objective:** To assess health insurance coverage, access to healthcare, and use of selected clinical preventive services recommended by USPSTF and ACIP by state, state Medicaid expansion status, expanded geographic region, and federal poverty level<br>**Date vintage:** 2014	Noninstitutionalized adults aged 18-64 years or older residing in the US	Of 299,157 respondents aged 18-64 years, 297,734 completed the question on insurance coverage:<br>▪ 82.4% reported having health insurance at the time of the interview<br>▪ Median estimated prevalence of insured adults at the time of the interview: 84.2%; however, this varied by state<br>▪ Estimated prevalence of insurance coverage:<br>– By age: 45-64 years (88%), 18-24 years (79.9%), and 25-44 years (79%)<br>– By sex: women (84%) and men (80.8%)<br>– Race: Hispanic (63.9%), non-Hispanic Black	NA	Patient barriers, with a focus on primary source of health insurance, Medicaid expansion status, income, and continuity of health insurance coverage

Author (date), title, US state	Vaccine type/s	Study details[a]	Inclusion/ exclusion criteria	Patient characteristics	Provider characteristics	Outcomes available
				(80%), non-Hispanic NHPI (63.9%), non-Hispanic multiple race (83.5%), non-Hispanic AIAN (84%), non-Hispanic other race (84.6%), non-Hispanic White (88%), and non-Hispanic Asian (88.4%)		

ACA = Affordable Care Act; ACIP = Advisory Committee on Immunization Practices; ACO = accountable care organization; ADVANCE = Accelerating Data Value Across a National Community Health Center Network; AIAN = American Indian and Alaska Native; APCD = All-Payer Claims Database; BMI = body mass index; BRFSS = Behavioral Risk Factor Surveillance System; CCAE = Commercial Claims and Encounters; CIIS = Colorado's Immunization Information System; EHR = electronic health record; FQHC = federally qualified health center; HPV = human papillomavirus; HZ = herpes zoster; IIS = Immunization Information System; IQR = interquartile range; MEPS = Medical Expenditure Panel Survey; MS = Medicare Supplemental; MSA = metropolitan statistical area; NA = not applicable; NHIS = National Health Interview Survey; NHPI = Native Hawaiian or Other Pacific Islander; NR = not reported; SD = standard deviation; Td = tetanus and diphtheria; Tdap = tetanus, diphtheria, and pertussis; US = United States; USPSTF = United States Preventive Services Task Force.

[a] Study details include the years the data were collected, study type, and methodology.

[b] Percentage calculated.

Table A6. Study Characteristics – Providers

Author (date), title, US state	Vaccine type/s	Study details[a]	Inclusion/ exclusion criteria	Patient characteristics	Provider characteristics	Outcomes available
**Author:** Cantu et al. (2020)<br>**Title:** *Longitudinal, multidisciplinary, resident-driven intervention to increase immunisation rates for Medicaid, low-income and uninsured patients*<br>**State:** Texas	Pneumococcal, influenza, varicella, HZ, Td	**Study type**: Longitudinal interventional study<br>**Data source:** Electronic medical record data from a primary care practice affiliated with the University of Texas Health San Antonio<br>**Objective:** Improve vaccination rates to an average of 76% of eligible Medicaid, low-income, and uninsured patients at an academic primary care practice<br>**Data vintage:** 2018	Eligible patients were 18 years of age or older and had Medicaid insurance, were low income, or were uninsured. Patients were excluded if they already met vaccination requirements for pneumococcal, influenza, varicella, HZ, and Td.	NR	NR	Provider barriers
**Author:** Lewis et al. (2020)	PCV13, PPSV23, Tdap, Td	**Study type**: advisory panel,	A random sample from the full universe of 1,367 federally	NA	The final sample was nationally representative of	Provider barriers

Author (date), title, US state	Vaccine type/s	Study details[a]	Inclusion/ exclusion criteria	Patient characteristics	Provider characteristics	Outcomes available
**Title:** *Vaccination capability inventory of community, migrant, and homeless health centers: a survey report*<br>**State:** National	booster, RZV, HPV	focus group and a survey<br>**Data source:** 319 health centers in the US and its territories<br>**Objective:**<br>To examine the inventory, workflow, capacity for. and barriers to the provision of routinely recommended for adult vaccinations<br>**Data vintage:** 2018	funded health centers. To have 95% ± 5% confidence and, assuming a 40% response rate, the project required that at least 762 surveys be distributed.		all health centers based on key characteristics such as size, urban or rural location, and the percentages of patients served with Medicaid and uninsured.	
**Author:** Granade et al. (2020)<br>**Title:** *State policies on access to vaccination services for low-income adults*<br>**State:** National	Influenza, Tdap, HPV, PPSV23, Hep A, Hep B	**Study type**: A public domain document review with supplemental semistructured telephone survey<br>**Data source:** Survey of Medicaid directors<br>**Objective:** To evaluate adult Medicaid	NR	NR	Medicaid program directors completed the survey.<br>Healthcare professional FFS reimbursement fee schedules were evaluated for 49 of 51 Medicaid programs; the remaining 2	Reimbursement for vaccine providers

Author (date), title, US state	Vaccine type/s	Study details[a]	Inclusion/ exclusion criteria	Patient characteristics	Provider characteristics	Outcomes available
		beneficiaries' access to adult immunization services through review of vaccination benefits coverage in Medicaid programs across the 50 states and the District of Columbia<br>**Data vintage:** June 2018-June 2019			programs, in Hawaii and Tennessee, are both under 100% MCO arrangements.	
**Author:** Goodman et al. (2019)<br>**Title:** *Billing and payment of commercial and Medicaid health plan adult vaccination claims in Michigan since the Affordable Care Act*<br>**State:** Michigan	Influenza, HPV, HZ, Pneumococcal, Hep A, Hep B	**Study type**: Data source analysis<br>**Data Source:** Blue Care Network of Michigan and Blue Cross Complete<br>**Objective:** To evaluate commercial and Medicaid adult vaccination claims and examine reasons for the nonpayment of	NR	NR	Michigan-based providers whose data were collected by Blue Care Network of Michigan and provided adult vaccinations	Provider barriers

Author (date), title, US state	Vaccine type/s	Study details[a]	Inclusion/ exclusion criteria	Patient characteristics	Provider characteristics	Outcomes available
		insurance claims after ACA<br>**Data vintage:** January 2014 through September 2015				
**Author:** Yarnoff et al. (2019)<br>**Title:** *Analysis of the profitability of adult vaccination in 13 private provider practices in the United States*<br>**State:** 9 states	All adult vaccines	**Study type**: Survey and time-motion study<br>**Data source:** 19 physician practices in 9 states<br>**Objective:** Understand how costs compared with payments for adult vaccinations in a sample of US physician practices<br>**Data vintage:** 2017	Practices were recruited in 4 cities, each in a different state; 2 cities from the top tercile of wages for medical professionals and 2 from the bottom were selected. Practices were eligible for the study if the practice self-reported providing 5 or more vaccinations per week.	NA	**Total N (physician practices) = 19**<br>▪ 3+ physicians in practice: 10 of 19<br>▪ Patients with Medicaid: 0%-20% (8 of 19 practices served no patients with Medicaid)<br>▪ Note that 3 small practices and 10 large practices were included in the final analysis	Provider focused barriers
**Author:** Lindley et al. (2018)<br>**Title:** *Vaccine financing and billing in practices serving*	Vaccines routinely recommended for adults ≥ 19 years other than	**Study type:** Survey (interviewer-administered)<br>**Data source:** Survey questions,	Personnel working in family medicine and general internal medicine practices who were considered knowledgeable about	NA	Personnel from 104 practices (out of 242 practices that were approached):	Provider barriers, with a focus on payments, purchasing practices, perceived profit,

Author (date), title, US state	Vaccine type/s	Study details[a]	Inclusion/ exclusion criteria	Patient characteristics	Provider characteristics	Outcomes available
adult patients: a follow-up survey **State:** Nationwide	influenza (excluding travel vaccines), seasonal influenza	which covered involvement in vaccine purchasing and billing on behalf of the practice, whether and how the practice bills Medicare Part D, percentage of the practice's annual budget allocated to adult vaccines, and experience with 6 payer types (private fee-for-service insurance, private preferred provider organizations, private health maintenance or managed care organizations, Medicaid, Medicare Part B, and Medicare Part D) **Objective:** To assess perceived payments and	vaccine financing and billing (as per information gathered in a previous survey conducted in 2013).		▪ Office managers or health administrators (52%) ▪ Billing staff (25%) ▪ Clinicians (14%)	claim denial, and financial concerns; knowledge, conflicting priorities/duration of medical appointments, and vaccine opportunities were mentioned as patient barriers in the background section

Author (date), title, US state	Vaccine type/s	Study details[a]	Inclusion/ exclusion criteria	Patient characteristics	Provider characteristics	Outcomes available
		profit from administering recommended adult vaccines, and vaccine purchasing practices among general internal medicine and family medicine practices in the US<br>**Data vintage:** January-June 2014				
**Author:** Hurley et al. (2017)<br>**Title:** *Primary care physicians' perspective on financial issues and adult immunization in the Era of the Affordable Care Act*<br>**State:** Nationwide	Zoster, Hepatitis B, Tdap, Hepatitis A, HPV, Meningococcal, Varicella, Td, PCV13, seasonal influenza, PPSV23, MMR	**Study type:** Survey<br>**Data source:** Survey administered to a national network of physicians, and conducted by the Vaccine Policy Collaborative Initiative<br>**Objectives:** To assess among general internists and family physicians shortly after the	National network of family physicians and general internist who spent at least half their time practicing primary care. This network was developed from the membership list of the ACP and the AAFP. Quota sampling was used to ensure that networks were similar with respect to region, urban/rural location, and practice setting (general	NA	Respondents included:<br>▪ 317 general internal medicine physicians:<br>– Mean age (SD): 52.9 (8.8)<br>– 62% male<br>– 46% urban, 41% suburban, and 13% rural<br>– 80% private practice, 17% hospital/	Provider barriers, with a focus on satisfaction with vaccine purchase and vaccine administration payments, vaccine coverage, vaccine affordability, knowledge of Medicare coverage, and anticipated changes in response to

Author (date), title, US state	Vaccine type/s	Study details[a]	Inclusion/ exclusion criteria	Patient characteristics	Provider characteristics	Outcomes available
		implementation of ACA:<br>1) Proportion of adult patients deferring or refusing vaccines because of cost and frequency of physicians not recommending vaccines for financial reasons<br>2) Satisfaction with reimbursement for vaccine purchase and administration by payer type<br>3) Knowledge of Medicare coverage of vaccines<br>4) Awareness of vaccine-specific provisions of ACA<br>**Data vintage:**<br>June-October 2013	internal medicine only).		clinic, and 4% HMO<br>– 97% providing vaccines to adults<br>▪ 236 family physicians:<br>– Mean age (SD): 52.9 (9.9)<br>– 56% male<br>– 25% urban, 46% suburban, and 29% rural<br>– 7% private practice, 24% hospital/ clinic, and 6% HMO<br>– 96% providing vaccines to adults<br>After excluding physicians that reported not providing adult vaccinations, the final cohort for	vaccine-specific provisions in ACA; patient barriers were also discussed but as reported by physicians: patients refusing or deferring vaccines due to financial issues

Author (date), title, US state	Vaccine type/s	Study details[a]	Inclusion/ exclusion criteria	Patient characteristics	Provider characteristics	Outcomes available
					analysis included 534 physicians (308 general internal medicine, and 226 family physicians)	
**Author:** Yarnoff et al. (2019)<br>**Title:** *Analysis of the profitability of adult vaccination in 13 private provider practices in the United States*<br>**State:** 9 states	All adult vaccines	**Study type**: Survey and time-motion study<br>**Data source:** 19 physician practices in 9 states<br>**Objective:** Describe a broad conceptual framework of vaccination and illustrate 4 potential determinants of influenza vaccination<br>**Data vintage:** 2017	Practices were recruited in 4 cities, each in a different state; 2 cities from the top tercile of wages for medical professionals and 2 from the bottom were selected. Practices were eligible for the study if the practice self-reported providing 5 or more vaccinations per week.	NA	**Total N (physician practices) = 19**<br>▪ 3+ physicians in practice: 10 of 19<br>▪ Patients with Medicaid: 0%-20% (8 of 19 practices served no patients with Medicaid)	Provider focused barriers
**Author:** Orenstein et al. (2007)<br>**Title:** *Financing immunization of*	All adult vaccines	**Study type**: Review<br>**Data source:** NA<br>**Objective:** Describe the	NA	NA	NA	▪ Patient general barriers<br>▪ Provider general barriers

Author (date), title, US state	Vaccine type/s	Study details[a]	Inclusion/ exclusion criteria	Patient characteristics	Provider characteristics	Outcomes available
adults in the United States<br>**State:** Nationwide		challenges to reducing vaccine-preventable morbidity and mortality in adults with a particular focus on vaccine financing issues<br>**Data vintage:** NA				

AAFP = American Academy of Family Physicians; ACA = Affordable Care Act; ACP = American College of Physicians; FFS = Medicaid fee-for-service; HMO = health maintenance organization; HPV = human papillomavirus; HZ = herpes zoster; MCO = managed care organization; MMR = measles-mumps-rubella vaccine; NA = not applicable; NR = not reported; PCV = pneumococcal conjugate vaccine; PPSV = pneumococcal polysaccharide vaccine; RZV = recombinant zoster vaccine; Td = tetanus and diphtheria; Tdap = tetanus, diphtheria, and pertussis; SD = standard deviation; US = United States.

[a] Study details include the years the data were collected, study type, and methodology.

Table A7. Outcomes for Patient-Focused Studies

Author (date), US state, vaccine	Patient cost barriers[a]	Patient perceived risks/benefits[b]	Other barriers[c]
**Author:** Kim et al. (2021)<br>**State:** 17 Medicaid expansion states and 19 non-expansion states<br>**Vaccine:** Influenza	NR	NR	**Vaccine access:**<br>An increase in the percentage of the population reporting having health insurance coverage was observed for Medicaid expansion states relative to non-expansion states.<br>**Vaccine opportunity:**<br>A reduction in length of time since last routine checkup was observed for Medicaid expansion states relative to non-expansion states.
**Author:** Hatch et al. (2021)<br>**State:** 10 Medicaid expansion states and 4 non-expansion states<br>**Vaccine:** HPV, influenza	NR	NR	**Vaccine access:**<br>Impact of Medicaid expansion was mixed across preventive services, suggesting that multilevel factors outside Medicaid expansion alone, such as coverage of preventive services, may impact overall receipt of preventive services.
**Author:** Osazuwa-Peters et al. (2020)<br>**State:** Nationwide<br>**Vaccine:** HPV	NR	NR	**Vaccine opportunity:**<br>Increased HPV vaccination rates post-ACA were accompanied by an increase in the rate and odds of regular physician visitation, suggesting that increased routine physician visit rates may contribute to better vaccination rates.
**Author:** Aris et al. (2020)<br>**State:** Nationwide	NR	NR	**Vaccine opportunity:**

Author (date), US state, vaccine	Patient cost barriers[a]	Patient perceived risks/benefits[b]	Other barriers[c]
**Vaccine:** Tdap, HZ, pneumococcal			Medicaid patients had fewer well-visits where adult vaccinations could be recommended.
**Author:** Inguva et al. (2020)<br>**State:** Mississippi<br>**Vaccine:** HPV	Vaccine cost was not considered a barrier by the authors because MS Medicaid beneficiaries below 19 years would receive VFC-funded vaccines, and HPV vaccine was covered by Mississippi Division of Medicaid for ages 19 years and older.	NR	**Education:**<br>There was a lack of educational activities to emphasize HPV vaccination as cancer prevention and the benefits of early vaccination.<br>**Vaccine access:**<br>No pharmacy claims for HPV vaccine identified, likely due to lack of pharmacists enrolled as VFC providers and inability to receive reimbursement through Mississippi Medicaid's administrative code for immunizations given at a pharmacy venue.<br>**Health plan type:**<br>Beneficiaries in managed care plans had lower completion odds relative to those in FFS plans, possibly due to lack of validated quality measure incorporating HPV vaccine completion to assess provider performance.
**Author:** Hurley et al. (2019)<br>**State:** Colorado<br>**Vaccine(s):** Influenza, Td/Tdap, pneumococcal	NR	NR	**Missed opportunities:**<br>Of the group aged 19-64 who were eligible for 2 vaccines at a given visit, only 5% received both vaccines.<br>**Standing orders:**<br>Only 1 out of 6 healthcare facilities had standing orders for all 3 vaccines.

Author (date), US state, vaccine	Patient cost barriers[a]	Patient perceived risks/benefits[b]	Other barriers[c]
			**Vaccination status documentation:**<br>IIS are currently preferentially populated with pediatric vaccination information.
**Author:** Wheldon et al. (2021)<br>**State:** National<br>**Vaccines:** HPV	NR	NR	**Knowledge/education:**<br>Unadjusted/adjusted ORs were reported for survey respondents to the following statements.<br>*I don't have enough information about the HPV vaccine to decide whether to get it or not (Agree vs. Neutral/Disagree)*<br>The OR (95% CI)/AOR (95%) for respondents in a Medicaid Expansion State vs. non-Medicaid Expansion State were 1.09 (0.79-1.49)/ 1.03 (0.74-1.44).<br>*I need more information about HPV vaccine efficacy (Agree vs. Neutral/Disagree)*<br>The OR (95% CI)/AOR (95%) for respondents in a Medicaid Expansion State vs. non-Medicaid Expansion State were 1.47 (1.06-2.04) [p < 0.05]/ 1.26 (0.89-1.80). In the health insurance category, public insurance was used as the reference.<br>The study also reported the perceived information needs for the overall population. Respondents were asked to respond to the following 2 statements.

Author (date), US state, vaccine	Patient cost barriers[a]	Patient perceived risks/benefits[b]	Other barriers[c]
			I don't have enough information about the HPV vaccine to decide whether to get it or not.<br>Responses:<br>▪ Agree: 400 (57.0%)<br>▪ Neutral: 177 (25.2%)<br>▪ Disagree: 125 (17.80%)<br>*I need more information about HPV vaccine efficacy.*<br>Responses:<br>▪ Agree/Strongly agree: 462 (65.8%)<br>▪ Neither: 145 (20.7%)<br>▪ Disagree/strongly disagree: 95 (13.5%)<br>Respondents were also asked which topics they would like to know more about before deciding to get vaccinated:<br>▪ Safety: 502 (82.7%)<br>▪ Effectiveness: 506 (83.4%)<br>▪ Good for me: 502 (82.7%)<br>▪ Doctor thinks important: 504 (83.0%)<br>▪ Side effects: 506 (83.4%)<br>▪ Risks: 513 (84.5%)
**Author:** Bloodworth et al. (2019)<br>**State:** Michigan<br>**Vaccine:** Influenza	**Cost-sharing:**<br>Cost-sharing has been documented as a substantial barrier to healthcare access (Solanki and Schauffler, 1999),[d] especially for low-income individuals.	NR	**Administrative and cultural:**<br>There is difficulty in enrolling and retaining low-income individuals due to factors such as transient housing and linguistic and cultural barriers.

Author (date), US state, vaccine	Patient cost barriers[a]	Patient perceived risks/benefits[b]	Other barriers[c]
**Author:** Hurley et al. (2017)<br>**State:** Nationwide<br>**Vaccine(s):** All adult vaccines	Shingles, hepatitis B, and HPV vaccines were most frequently reported as having patients deferring or refusing vaccines due to financial reasons. For example, over half of physicians reported that 20% or more of their patients in a typical month deferred or refused shingles vaccination for financial reasons.	NR	NR
**Author:** Stewart et al. (2014)<br>**Title:** *Vaccination benefits and cost-sharing policy for noninstitutionalized adult Medicaid enrollees in the United States* **State:** Nationwide [2 programs (WV, WI) declined to participate and 7 (IL, KS, NH, NC, OH, PA, RI) did not respond]<br>**Vaccine(s):** All adult vaccines	In 2012, adult vaccine copayments ranged from $0.50 to $3.40 for Medicaid beneficiaries.	NR	**Vaccine access:**<br>As of 2012, 15 out of 51 programs did not cover all ACIP-recommended adult vaccines, and only 17 programs covered all ACIP-recommended adult vaccines while also prohibiting copayments.
**Author:** New et al. (2018)<br>**State:** California<br>**Vaccines:** Tdap	NR	NR	**Vaccine opportunity:**<br>Mothers who were offered Tdap vaccination onsite during a routine prenatal appointment were more likely to be vaccinated than mothers who were referred offsite for vaccination:<br>▪ 24 of 26 women who reported receiving Tdap were vaccinated at their prenatal care provider's office.

Author (date), US state, vaccine	Patient cost barriers[a]	Patient perceived risks/benefits[b]	Other barriers[c]
			▪ 25 percent of mothers who did not received Tdap did not receive a recommendation or referral offsite from their provider, 23% were referred offsite but did not receive Tdap, 18% were deferred by their prenatal care provider for reasons not considered by ACIP to be a contraindication, and 8% did not receive prenatal care.<br>**Healthcare insurance:**<br>Mothers insured by Medicaid were less likely to receive Tdap than mothers with private insurance, regardless of onsite availability of the vaccine:<br>▪ 54% of mothers with private insurance received Tdap on time, compared with 18% of mothers with Medicaid insurance<br>▪ Mothers insured by Medicaid were significantly less likely than those with private insurance to receive prenatal Tdap: RR = 0.4 (95% CI, 0.2-0.8)<br>▪ Mothers insured by Medicaid were less likely than those with private insurance to receive prenatal Tdap in the appropriate timeframe, even when Tdap was stocked onsite: RR = 0.5 (95% CI, 0.3-1.1)<br>**Vaccine access:**<br>Mothers whose prenatal care provider stocked Tdap onsite were significantly more likely to be vaccinated than mothers

Author (date), US state, vaccine	Patient cost barriers[a]	Patient perceived risks/benefits[b]	Other barriers[c]
			whose prenatal care provider did not stock Tdap: RR = 3.3 (95% CI, 1.9-5.5).<br>**Personal reasons:**<br>Twenty percent of mothers who did not receive Tdap reported refusing the vaccine for personal reasons.
**Author:** Brown et al. (2018)<br>**State:** Texas<br>**Vaccines:** Influenza, pneumonia, HPV	NR	NR	**Lack of time:** Patients lacked time to visit a center that was administering vaccinations that are too far away.<br>**Indecision:** Indecision regarding whether or not to get the vaccinations.
**Author:** Lindley et al. (2018)<br>**State:** nationwide<br>**Vaccines:** Vaccines routinely recommended for adults ≥ 19 years other than influenza (excluding travel vaccines), seasonal influenza	Medicaid was the only payer for which the majority of respondents (60%) reported payment less than purchase price.<br>Less than one-third of respondents seeing patients in HMO/ MCOs, Medicaid, or Medicare reported profiting on vaccinations	NR	**Knowledge:**<br>Not knowing that vaccines are needed<br>**Conflicting priorities/duration of medical appointments:**<br>Other issues taking precedence during brief medical visits<br>**Vaccine opportunity:**<br>Physicians not recommending vaccinations
**Author:** Stoecker et al. (2017)<br>**State:** Nationwide (49 states, excluding the District of Columbia and Florida)<br>**Vaccines:** Influenza	**Coverage benefits:**<br>Providing Medicaid coverage benefits for influenza vaccine led to a statistically significant increase in coverage levels for all adults:<br>▪ 3.6 percentage point higher likelihood of reporting being	**Cost-sharing:**<br>The impact of prohibiting cost-sharing on influenza vaccination coverage was not statistically significant across all patient groups.	NR

Author (date), US state, vaccine	Patient cost barriers[a]	Patient perceived risks/benefits[b]	Other barriers[c]
	vaccinated ($p < 0.01$) for all adults aged 19-64 years<br>▪ 6.2 percentage point higher likelihood of reporting being vaccinated ($p < 0.01$) for all adults aged 50-64 years.<br>There was no statistically significant difference in Medicaid-enrolled pregnant women aged 19-64 years ($p = 0.65$), and in Medicaid-enrolled parents aged 19-64 years ($p = 0.99$)<br>**Copayment:**<br>Medicaid copayment charges negatively impacted influenza vaccination levels for all patient groups:<br>▪ All adults aged 19-64 years: a $1 increase in copayments led to a 0.6 percentage point decline in vaccination coverage ($p = 0.014$)<br>▪ All adults aged 50-64 years: a $1 increase in copayments led to a 1.1 percentage point decline in vaccination coverage ($p = 0.014$)<br>The effect was larger in Medicaid-enrolled pregnant women aged 19-64 years (6.2		

Author (date), US state, vaccine	Patient cost barriers[a]	Patient perceived risks/benefits[b]	Other barriers[c]
	percentage point decline), and in Medicaid-enrolled parents aged 19-64 years (1.4 percentage point decline); however, in both cases, the results were not statistically significant.		
**Author:** Yue et al. (2018)<br>**State:** Nationwide<br>**Vaccine:** Influenza	NR	NR	**Access to care:**<br>The study reported the adjusted difference in differences estimates of the impacts of Medicaid expansions on outcome variables in the overall sample and by racial/ethnic groups.<br>For the low-income, nonelderly population, Medicaid expansion was associated with a 7.10 percentage point increase in health insurance coverage. However, Medicaid Expansion was associated with a 0.68 percentage point reduction in receiving a flu shot for the overall population.<br>Results by race/ethnicity:<br>▪ Non-Hispanic White: 2.20<br>▪ Non-Hispanic Black: −6.24<br>▪ Hispanic: −5.12<br>▪ Non-Hispanic other: −0.85<br>None of the differences were statistically significant.
**Author:** Beckett et al. (2015)<br>**State:** Nationwide	NR	NR	**Living alone:**<br>Seniors who lived alone reported worse influenza (in past year) and pneumococcal

Author (date), US state, vaccine	Patient cost barriers[a]	Patient perceived risks/benefits[b]	Other barriers[c]
**Vaccine(s):** Influenza, pneumococcal			(ever) immunization rates relative to those who did not live alone, with disparities between the 2 groups greater for males than females.
**Author:** Orenstein et al. (2007)<br>**State:** Nationwide<br>**Vaccine(s):** All adult vaccines	As of 2007, 4.2% of adults aged 18-64 years were both uninsured and below 200% of the poverty line, creating financial challenges for paying for vaccines out of pocket.	NR	**Vaccine access:**<br>Low levels of Section 317 funding compared with estimated needs may reduce access to recommended vaccines for uninsured and underinsured adults.
**Author:** Hawkins et al. (2021)<br>**State:** Maine, New Hampshire, Massachusetts<br>**Vaccine(s):** HPV	NR	NR	**Vaccine access:**<br>Implementation of ACA policies and the ACIP recommendation for routine vaccination of males were associated with increased uptake of HPV vaccine among males with Medicaid.<br>**Age:**<br>Adults (aged 20-26 years) had lower vaccine initiation rates relative to children and adolescents, and initiation rates decreased over the study period for this age group.
**Author:** Okoro et al. (2017)<br>**States:** Nationwide (50 states and the District of Columbia)<br>**Vaccines:** Influenza	NR	NR	**Primary source of insurance:**<br>The estimated prevalence of influenza vaccination was lowest among adults with no primary source of insurance (17.8 [95% CI, 17.0-18.6; crude] and 18.0 [95% CI, 17.2-18.6; age standardized]) and generally similar for publicly (38.6 [95% CI,

Author (date), US state, vaccine	Patient cost barriers[a]	Patient perceived risks/benefits[b]	Other barriers[c]
			37.7-39.4; crude] and 37.4 [95% CI, 36.5-38.2; age standardized]) and privately insured adults (37.9 [95% CI, 37.4-38.3; crude], and 36.7 [95% CI, 36.3-37.2; age standardized]).<br>Estimates by Medicaid status:<br>▪ Medicaid expansion:<br>– No primary source: 18.2 (95% CI, 16.9-19.5; crude), and 18.5 (95% CI, 17.3-19.7; age standardized)<br>– Public insurance: 36.6 (95% CI, 35.5-37.7; crude), and 35.6 (95% CI, 34.4-36.8; age standardized)<br>– Private insurance: 37.9 (95% CI, 37.3-38.5; crude), and 36.7 (95% CI, 36.1-37.3; age standardized)<br>▪ Medicaid non-expansion:<br>– No primary source: 17.4 (95% CI, 16.4-18.4; crude), and 17.6 (95% CI, 16.6-18.6; age standardized)<br>– Public insurance: 41.6 (95% CI, 40.4-42.9; crude), and 40.1 (95% CI, 38.7-41.4; age standardized)<br>– Private insurance: 37.8 (95% CI, 37.2-38.4; crude), and 36.7 (95% CI, 36.1-37.4; age standardized)<br>**Medicaid expansion and income:**<br>The estimated prevalence of influenza vaccination was comparable between Medicaid expansion and non-expansion states:

Author (date), US state, vaccine	Patient cost barriers[a]	Patient perceived risks/benefits[b]	Other barriers[c]
			▪ Medicaid expansion: 34.3 (95% CI, 33.8-34.8) (crude), and 33.6 (95% CI, 33.1-34.1) age standardized<br>▪ Medicaid non-expansion: 33.4 (95% CI, 32.9-33.8) (crude), and 32.8 (95% CI, 32.3-33.2)<br>In Medicaid expansion states, the estimated prevalence of influenza vaccination was similar among adults with household income < 100% of FPL (29.9%), and those with household income ≥ 100%-400% of FPL (30.2%); in non-expansion states, the estimated prevalence of influenza vaccination increased with increasing income (crude and age-standardized estimates are provided in Table 5 of the paper)<br>**Continuity of health insurance coverage:**<br>Overall and by state Medicaid expansion status, the estimated prevalence of influenza vaccination was highest among adults who were continuously insured, followed by those who had a gap in insurance coverage, and lowest among those who were without health insurance for more than 12 months:<br>▪ Overall estimates:<br>– Continuously insured in the preceding 12 months: 39.1 (95% CI, 38.7-39.5; crude), and 37.9 (95% CI, 37.5-38.3; age standardized)

Author (date), US state, vaccine	Patient cost barriers[a]	Patient perceived risks/benefits[b]	Other barriers[c]
			– Gap in insurance in the preceding 12 months: 26.1 (95% CI, 25.1-27.1; crude), and 26.5 (95% CI, 25.5-27.4; age standardized)<br>– No health insurance in the preceding 12 months: 14.7 (95% CI, 13.8-15.6; crude), and 14.9 (95% CI, 14.1-15.8; age standardized)<br>Estimates by Medicaid status:<br>▪ Medicaid expansion:<br>– Continuously insured in the preceding 12 months: 38.7 (95% CI, 38.2-39.3; crude), and 37.5 (95% CI, 36.9-38.1; age standardized)<br>– Gap in insurance in the preceding 12 months: 26.1 (95% CI, 24.7-27.5; crude), and 26.3 (95% CI, 25.0-27.7; age standardized)<br>– No health insurance in the preceding 12 months: 14.4 (95% CI, 13.1-15.9; crude), and 14.7 (95% CI, 13.4-16.2; age standardized)<br>▪ Medicaid non-expansion:<br>– Continuously insured in the preceding 12 months: 39.6 (95% CI, 39.0-40.2; crude), and 38.4 (95% CI, 37.8-39.0; age standardized)<br>– Gap in insurance in the preceding 12 months: 26.2 (95% CI, 24.8-27.6; crude), and 26.6 (95% CI, 25.2-28.0; age standardized)<br>– No health insurance in the preceding 12 months: 14.9 (95% CI, 13.8-16.1;

Author (date), US state, vaccine	Patient cost barriers[a]	Patient perceived risks/benefits[b]	Other barriers[c]
			crude), and 15.1 (95% CI, 14.0-16.2; age standardized)

ACA = Affordable Care Act; ACIP = Advisory Committee on Immunization Practices; ACO = accountable care organization; AOR = adjusted odds ratio; CHC = community health centre; CI = confidence interval; FFS = Medicaid fee-for-service; FPL = federal poverty level; HMO/MCO = private health maintenance or managed care organizations; HPV = human papillomavirus; HZ = herpes zoster; IIS = Immunization Information System; MS = Medicare Supplemental; NA = not applicable; NR = not reported; OR = odds ratio; RR = risk ratio; Td = tetanus and diphtheria; Tdap = tetanus, diphtheria, and pertussis; US = United States; VFC = Vaccines for Children.

[a] Includes direct costs, indirect costs, perceived costs, and any other costs.

[b] Includes actual costs, perceived costs, and any other costs.

[c] For example, accurate medical record keeping and knowledge barriers.

[d] Solanki G, Schauffler HH. Cost-sharing and the utilization of clinical preventive services. Am J Prev Med. 1999;17(2):127-33.

Table A8. Outcomes for Provider-Focused Studies

Author (date), US state, vaccine	Provider cost barriers[a]	Provider risks/benefits[b]	Other barriers[c]
**Author:** Cantu et al. (2020)<br>**State:** Texas<br>**Vaccines:** Pneumococcal, influenza, HZ, Td	NR	NR	The first intervention cohort identified several challenges during immunization-related workflows, including staff unfamiliarity with vaccination schedule, lack of standing orders, confusion on tasks, difficulty accessing the online state vaccination registry, and limited time to update a patient's vaccination history during the intake process.
**Author:** Lewis et al. (2020)<br>**State**: National<br>**Vaccines:** PCV13, PPSV23, Tdap, Td booster, RZV, HPV	Some providers did not stock HZ due to the perceived high costs and low reimbursement for HZ.<br>However figure 2 in the paper demonstrated that, for Medicaid reimbursement, 60% of centers felt dose reimbursement was adequate and 63% perceived administration reimbursement to be adequate for vaccinations.	NR	The background of the study also indicated that the following could be barriers for vaccination for both patients and providers:<br>▪ Missed vaccination opportunities<br>▪ Miscommunication<br>▪ Health literacy<br>▪ Culture<br>▪ Social determinants of health
**Author:** Granade et al. (2020)<br>**State:** National<br>**Vaccine:** Influenza, Tdap, HPV, PPSV23, Hep A, Hep B	The study reported wide variance in the rates of reimbursement for vaccine administration (4 vaccine codes). Reimbursement was the same across the 4 codes in 11 of 49 programs, ranging from $3.72 in South Carolina to $20.80 in New Mexico. The programs with the highest reimbursement	NR	NR

Author (date), US state, vaccine	Provider cost barriers[a]	Provider risks/benefits[b]	Other barriers[c]
	amounts to healthcare professionals under FFS arrangements for a single injected vaccine administration (CPT code 90471) were Alaska ($28.18), Arizona ($22.32), and Nevada ($22.22). The programs with the lowest reimbursement amounts to healthcare professionals were South Carolina ($3.72), California ($4.46), Alabama ($5.00), and New Hampshire ($5.00).<br>There was also disparity in the reimbursement for vaccine purchase. Among all ACIP-recommended adult immunizations, median reimbursement was highest for the 9vHPV vaccine (CPT code 90651) at $204.87. The 9vHPV vaccine also demonstrated the largest per-dose reimbursement range, from $5.27 in Missouri to $491.38 in Mississippi. Median reimbursement was below the private sector price reported by manufacturers to the CDC for 7 of 13 ACIP-recommended adult vaccines, with the largest observed disparities for the		

Author (date), US state, vaccine	Provider cost barriers[a]	Provider risks/benefits[b]	Other barriers[c]
	varicella, 9vHPV, and Tdap vaccines.		
**Author:** Goodman et al. (2019)<br>**State:** Michigan<br>**Vaccine:** Influenza, HPV, HZ, Pneumococcal, Hep A, Hep B, Tdap	The study introduction noted that a 2012 national survey reported concerns among providers that payment was a major barrier to offering adult vaccinations. This was not specific to the Medicaid population though, and was a wider problem for all payer types.<br>For Medicaid claims in the pharmacy setting, the study reported that 100% of vaccine claims were paid, compared with 91.6% paid claims in the physician's office. For Medicaid, the most common reasons for nonpayment were contractual arrangements (52.3%), health plan eligibility (37.0%), no authorization (4.9%), and other claim submission error (4.4%).<br>Medicaid generally had lower rates of payment than commercial insurance, often due to complexities related to contractual arrangements or when and how coverage applies to an individual patient.	NR	**Medicaid eligibility:**<br>Some Medicaid providers could be seeing nonpayment resulting from frequent changes in patients' Medicaid eligibility.<br>**Code errors:**<br>Another reason for nonpayment is coding and billing errors on submitted claims.

Author (date), US state, vaccine	Provider cost barriers[a]	Provider risks/benefits[b]	Other barriers[c]
**Author:** Hurley et al. (2017)<br>**State:** Nationwide<br>**Vaccines:** All adult vaccines	**Perceived patient cost barriers:**<br>Over one-third of physicians reported they had not recommended vaccines ‘frequently’ or ‘sometimes’ because they either thought the patient’s insurance would not cover it or that the patient could receive the vaccine more affordably elsewhere; nearly 25% had not recommended vaccination believing that the patient could not afford it.<br>**Reimbursement dissatisfaction:**<br>Over half of physicians who were aware of their Medicaid reimbursements for vaccine purchase and administration were very or mostly dissatisfied with reimbursement amounts.	NR	**Awareness of ACA provisions:**<br>Physicians lacked knowledge of vaccine-specific provisions of the ACA, resulting in 71% reporting that their practice would not change the number of ACIP-recommended adult vaccines their practice stocks.
**Author:** Orenstein et al. (2007)<br>**State:** Nationwide<br>**Vaccine(s):** All adult vaccines	**Reimbursement:**<br>Uncertainty about if Medicaid reimbursement is adequate to cover costs of vaccines and nonvaccine costs (storage, record keeping, administration), as well as a reasonable return on investment.	NR	**NR**

Author (date), US state, vaccine	Provider cost barriers[a]	Provider risks/benefits[b]	Other barriers[c]
**Author:** Yarnoff et al. (2019)<br>**State:** 9 states<br>**Vaccines:** All adult vaccines	**Reimbursement:**<br>Median Medicaid reimbursements for vaccine administration ($13.56) were significantly lower than median Medicare ($20.18) and private payer ($24.57) reimbursements. Medicaid vaccine administration reimbursement were lower than costs for vaccine administration for OB-GYN practices but were higher than costs for family medicine and internal medicine practices. 5 of 14 vaccines had lower median Medicaid reimbursement than median price per dose.	NR	Differences between dose price and payment may make it difficult for practices to offer all vaccines. Practices may not stock all recommended vaccines because they are given infrequently
**Author:** Hurley et al. (2019)<br>**State:** Colorado<br>**Vaccine(s):** Influenza, Td/Tdap, pneumococcal	**NR**	NR	**Standing orders:**<br>Only 4 out of 6 healthcare facilities had standing orders for all 3 vaccines.<br>**Vaccination status documentation:**<br>IIS are currently preferentially populated with pediatric vaccination information.

ACA = Affordable Care Act; ACIP = Advisory Committee on Immunization Practices; CDC = Centers for Disease Control and Prevention; CMS = Centers for Medicare and Medicaid Services; CPT = Current Procedural Terminology; EMR = electronic medical record; FFS = Medicaid fee-for-service; Hep = hepatitis; HPV = human papillomavirus; HZ = herpes zoster; IIS = Immunization Information System; MMR = measles-mumps-rubella vaccine; NR = not reported; OB-GYN = obstetrics and gynecology; PCV = pneumococcal conjugate vaccine; PPSV = pneumococcal polysaccharide vaccine; RZV = recombinant zoster vaccine; Td = tetanus and diphtheria; Tdap = tetanus, diphtheria, and pertussis; US = United States.

[a] Includes direct costs, indirect costs, perceived costs, and any other costs.

[b] Includes actual costs, perceived costs, and any other costs.

[c] For example, accurate medical record keeping.
